# Engineered nanoparticles for precise targeted drug delivery and enhanced therapeutic efficacy in cancer immunotherapy

**DOI:** 10.1016/j.apsb.2024.05.010

**Published:** 2024-05-13

**Authors:** Xueqiang Peng, Jianjun Fang, Chuyuan Lou, Liang Yang, Shaobo Shan, Zixian Wang, Yutong Chen, Hangyu Li, Xuexin Li

**Affiliations:** a Department of General Surgery, The Fourth Affiliated Hospital, China Medical University, Shenyang 110032, China; b Department of Ophthalmology, Xi'an People's Hospital (Xi'an Fourth Hospital), Xi'an 710004, China; c Department of Neurosurgery, Beijing Tiantan Hospital, Capital Medical University, Beijing 10050, China; d Department of Medical Oncology, Sun Yat-sen University Cancer Center, State Key Laboratory of Oncology in South China, Collaborative Innovation Center of Cancer Medicine, Guangzhou 510060, China; e Department of Pathology, Medical College, Jinan University, Guangzhou 510632, China; f Department of Physiology and Pharmacology, Karolinska Institutet, Stockholm SE-17177, Sweden

**Keywords:** Engineered nanoparticles, Cancer immunotherapy, Drug delivery, Blood–brain barrier, Tumor-barrier, Nanomedicine, Nanomaterial, Cancer

## Abstract

The advent of cancer immunotherapy has imparted a transformative impact on cancer treatment paradigms by harnessing the power of the immune system. However, the challenge of practical and precise targeting of malignant cells persists. To address this, engineered nanoparticles (NPs) have emerged as a promising solution for enhancing targeted drug delivery in immunotherapeutic interventions, owing to their small size, low immunogenicity, and ease of surface modification. This comprehensive review delves into contemporary research at the nexus of NP engineering and immunotherapy, encompassing an extensive spectrum of NP morphologies and strategies tailored toward optimizing tumor targeting and augmenting therapeutic effectiveness. Moreover, it underscores the mechanisms that NPs leverage to bypass the numerous obstacles encountered in immunotherapeutic regimens and probes into the combined potential of NPs when co-administered with both established and novel immunotherapeutic modalities. Finally, the review evaluates the existing limitations of NPs as drug delivery platforms in immunotherapy, which could shape the path for future advancements in this promising field.

## Graphical abstract

This paper reviews recent advances in various nanoparticle-enhanced tumor immunotherapy strategies as delivery vehicles for immune agents.

**Figure:**
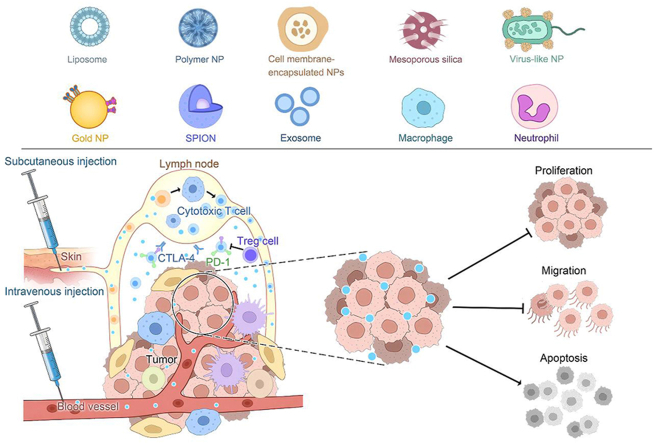

## 1 Introduction

Immunotherapy represents a groundbreaking paradigm in oncological therapeutics, leveraging the host's immune system to distinguish and eradicate malignant cells^1^^,^^2^. As a therapeutic modality, it offers a stark contrast to conventional treatments such as chemotherapy and radiotherapy, which frequently inflict considerable collateral harm on healthy tissues. However, the potency of immunotherapy is recurrently hampered by a complex interplay of physiological and pathological impediments, in addition to the immunosuppressive environment endemic to the tumor milieu itself. Traditional immunotherapeutic agents, encompassing monoclonal antibodies, immune checkpoint inhibitors, cytokines, and immune agonists, encounter formidable challenges in targeting this intricate tumor microenvironment (TME). These obstacles arise from the dual presence of the abovementioned barriers and the counteractive measures enacted by the tumor's suppressive ecosystem, significantly curtailing their therapeutic efficacy^3^. For instance, even the most advanced immune checkpoint inhibitors currently attain a success rate of approximately 30% in oncological interventions, accentuating the urgent need to enhance the precision and effectiveness of tumor immunotherapy delivery mechanisms^4^. In this context, NPs have emerged as a potent catalyst for reshaping the landscape of cancer immunotherapy. NPs possess the inherent ability to overcome numerous limitations associated with traditional pharmaceutical delivery modalities^5^^,^^6^. They hold the potential to enhance therapeutic drug targeting, boost bioavailability, facilitate immune evasion, and reduce adverse toxicity. Due to their intrinsic versatility, adaptability, and amenability to modification, NPs present a promising pathway to address the challenges of various cancer subtypes. Their utilization could mark a crucial turning point in optimizing immunotherapy, paving the way for more effective and patient-specific cancer treatment modalities.

In this systematic review, we explore the growing potential of NPs as innovative solutions to the intrinsic hurdles plaguing immunotherapeutic applications. We scrutinize NP variables to devise strategies for enhanced neoplastic cell targeting and tactics to bolster therapeutic outcomes. Moreover, our analysis extends into the physiological and pathological impediments that compromise the effectiveness of immunotherapy, and we illuminate the synergistic possibilities arising from the integration of NPs with both traditional and avant-garde immunotherapeutic strategies. Conclusively, the review critically evaluates the present limitations of NPs as drug delivery conduits in immunotherapy, offering a trajectory for prospective advancements in this dynamic and promising domain.

## 2 Diverse spectrum of nanoparticle modalities in oncology

The origin of nanoscience and nanotechnology can be ascribed to Richard Feynman's seminal lecture in 1959, "There is Plenty of Room at the Bottom," which shed light on the complexity of biological materials at the nanoscale^7^. Nanoparticles, synthesized from many substances, including lipids, polymers, and metal-based materials, have been vigorously adopted across various sectors^8^, ^9^, ^10^. These encompass Biomedicine, Electronics, Environmental Remediation, Catalysis, Material Science, Food and Agriculture, and Cosmetics^11^^,^^12^. The following section categorizes nanoparticles into three main classes: organic, inorganic, and biomimetic NPs. We highlight the applications of these three classes of NPs in these different disciplines and their therapeutic implications in oncology for a comprehensive analysis.

### 2.1 Organic NPs

#### 2.1.1 Liposomal NPs

**Figure 1:**
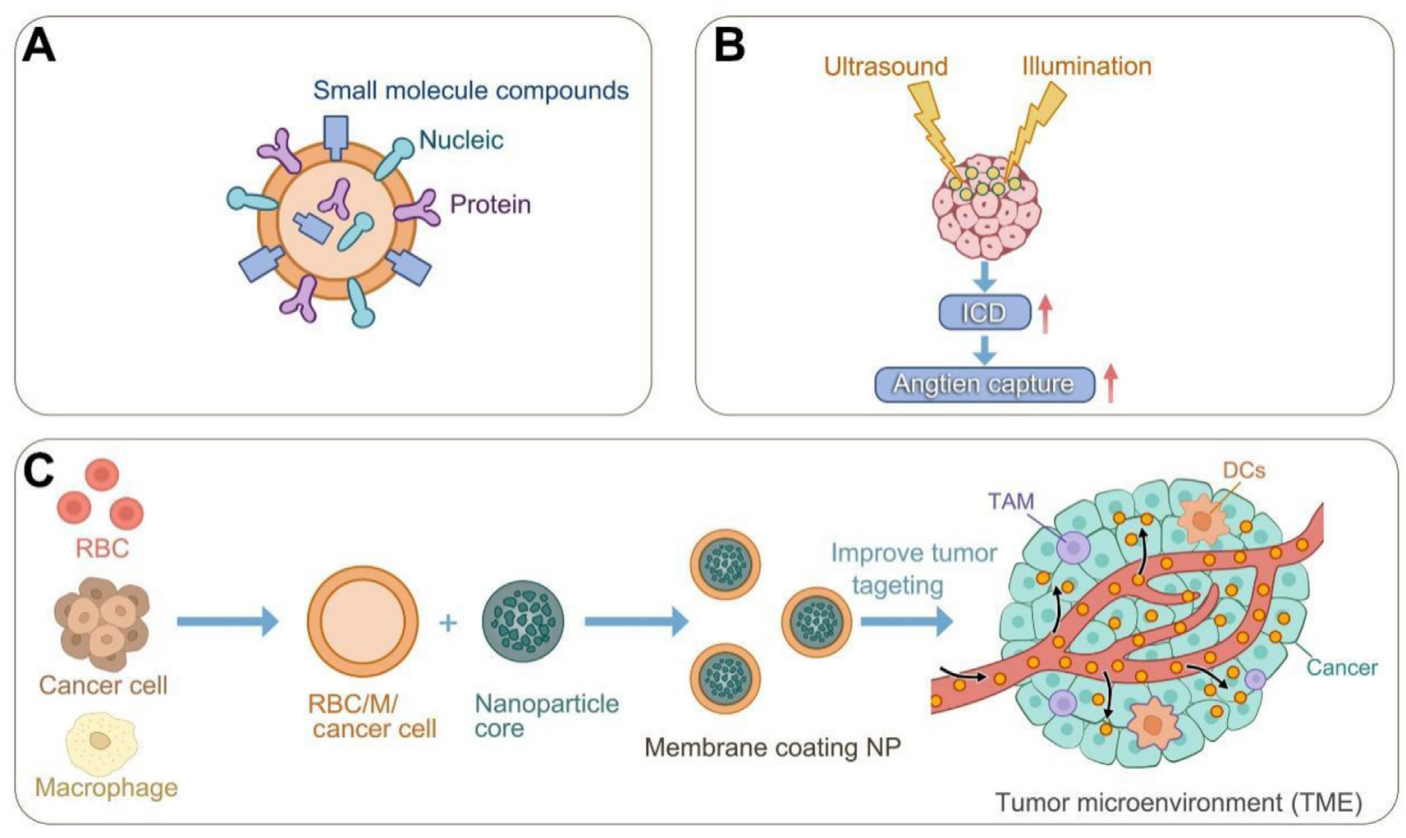
Designing liposomes to carry drugs for tumor immunotherapy. (A) Liposomes can carry a variety of therapeutic substances on their surface and inside. (B) Liposomes can be designed to enhance the efficacy of tumor immunotherapy in combination with PTD, SDT, and PPT. (C) Detachment of cell membranes from various cells (*e*.*g*., erythrocytes, macrophages, tumor cells) by ultrasonication, freeze-thaw, hypotonic lysis buffer, extrusion, etc. Wrapping the detached cell membrane into the liposome core surface by microfluidic electroporation, ultrasound/extrusion, and extrusion/electroporation, improving the ability of liposomes to target tumors.

Liposomal NPs are mainly composed of four lipid components, including phospholipids, especially 2-stearoyl-*sn*-glycerol-3-phosphocholine or 1,2-dioleoyl-*sn*-glycerol-3phosphatidylethanolamine (DOPE), and auxiliary lipids^13^^,^^14^. These are the first nanoscale therapeutic particles to be approved for the clinical treatment of oncology. In 1964, first observed the morphological structure of phospholipid vesicles under an electron microscope, followed by Bangham A. in 1965, who found that phospholipid molecules can spontaneously form closed bilayer vesicles in water with a composition and structural organization similar to that of cell membranes^15^^,^^16^. Local liposomes can be artificially assembled by various techniques, including, but not limited to, membrane hydration, ethanol injection, and emulsification^17^^,^^18^. The affinity of different parts of the vesicles can be utilized to encapsulate hydrophilic or lipophilic therapeutic substances or immunotherapeutic agents in aqueous phases or bilayers using methods such as co-incubation, ultrasonication, and microfluidics and introduce liposomes into the field of drug delivery systems^19^^,^^20^. Their strong biocompatibility has facilitated the understanding of liposomes, the ability to shield against therapeutic payloads, and adverse effects on the host (Fig. 1A). Notably, native liposome nanovesicles lack specialized functions, manifesting in suboptimal tumor-targeting efficiency and accelerated efflux from the circulatory system. Therefore, their synthetic modification is essential^21^. Common modifications include anti-adhesive or anti-phagocytic surface coatings, therapeutic drug loading, and imaging dyes for *in vivo* regression tracking. Conversion of pristine liposomal nanovesicles into functionalized NPs is critical for tumor diagnosis and therapy. Enzymatic reactions catalyze the release of drugs from these NPs under specific circumstances, such as teletherapy, ultrasound irradiation, light exposure, and disease-specific pH environments^22^ (Fig. 1B and C). At this stage, the well-known liposomal formulation is Doxil®, approved by the U.S. Food and Drug Administration (FDA) as a nanomedicine formulation for use in breast, ovarian, and other solid tumors^23^. In addition, liposome-encapsulated Zoerythromycin (DaunoXome) has been approved for clinical AIDS-related Kaposi sarcoma treatment^24^.

#### 2.1.2 Polymer NPs

Although liposomal NPs have been widely used as nanoscale carriers in clinical applications, their costly cryogenic storage requirements and their inherent thermal instability pose significant barriers to their application. Therefore, a growing interest in polymeric NPs, capable of withstanding autoclaving, lyophilization, and long-term storage, has emerged as a new direction in nanocarrier research^25^^,^^26^. Polymer NPs can be categorized into natural polymer NPs (*e*.*g*., peptides, albumin, gelatin, or chitosan) and synthetic polymer NPs (polyethylene PEG, poly (lactic acid)-glycolic acid copolymer (PLGA), poly (lactic acid) (PLA), and poly(caprolactone)) according to the source of raw materials used for their synthesis and their biological properties are different, with the natural polymers being biocompatible and biodegradable, while the synthetic polymer NPs are more highly targeted^27^. Polymeric NPs can be further categorized into nanocapsules, nanospheres, and dendritic polymers based on their diverse structural forms^28^. Structural differences result in different cargo loading locations, with nanocapsules typically encapsulating the "cargo" in an aqueous core and either the surface or the interior of the nanorods loading the cargo^29^^,^^30^. At the same time, dendritic polymers exhibit high surface functionality, with an exponential increase in the number of surface groups with the addition of each generation. Emulsion polymerization, solvent evaporation, salting, dialysis, and supercritical fluid techniques are the main methods of polymer nanoparticle synthesis^31^. In 2005, Abraxane became approved for clinical use as a delivery vehicle^32^. In addition to monomer polymer NPs, there has been an increasing interest in forming polymer micelles by self-assembling two or more monomer amphiphilic polymers^33^. In aqueous media, when the concentration of amphiphilic polymers is equal to or greater than the critical micelle concentration, micelles with a size of about 10–100 nm and a morphology that is usually spherical can be self-assembled^34^. The micelles have a hydrophobic core and a hydrophilic shell. The most commonly used micelles include A-B diblock and A-B-A triblock, where A and B represent hydrophilic and hydrophobic blocks, respectively. Using surface coupling techniques, these carriers can be modified by tumor-targeting therapeutic molecules, thereby expanding their medicinal capabilities^35^. However, the main drawbacks of polymeric NPs as tumor immuno-drug delivery platforms are poor stability, large size, and potential toxicity^36^.

### 2.2 Inorganic NPs

#### 2.2.1 Mesoporous silica

Mesoporous silica has attracted significant interest from the scientific community as an inorganic functional nanocarrier platform for cancer therapy due to its superior biocompatibility, wide range of surface domains, tunable particle size, diverse structural conformation, and direct available modifications^37^. The high silica concentration in supporting tissues *in vivo* makes it an endogenous biomaterial, which may underlie its excellent biocompatibility. By controlling the reaction conditions (*e*.*g*., reaction temperature, pH, surfactant concentration), silica can be prepared into multilayered structures of nanoscale mesoporous silica particles in a variety of shapes (hollow spheres, tubes, gyroscopes, spirals, etc.)^38^. Silica carriers are generally considered non-toxic and are usually degraded by hydrolysis through siloxane bonds in the silica matrix, which are then excreted *via* the fecal or urinary route^39^. More than 90% of the carrier is usually excreted within a week of administration. Mesoporous silica can self-polymerize into micelles, forming inorganic hybrids on its surface. Subsequent removal of the templated silica surfactant by solvent extraction and calcination allows the formation of pores that act as drug sequestration^40^. Currently, mesoporous silica serves as a drug delivery conduit for a range of biologically active entities, including chemical drugs, proteins, and peptides^41^. Surface modifications of components such as peptides, folic acid, mannose, and transferrin have been reported to lead to active tumor targeting^42^. However, multiple immune cell-mediated phagocytosis results in a shorter circulating half-life for conventional mesoporous silica, and modifications to their surface modifications are necessary^43^.

#### 2.2.2 Gold nanoparticles

Gold nanoparticles (AuNPs) have the advantages of low cytotoxicity, simple synthesis, biocompatibility, and *in vivo* stability, which make them an ideal base material for tumor-targeted therapy. Gold nanoparticles are essentially inert and have low toxicity, and ingestion of AuNPs in the body causes no adverse effects. AuNPs can enhance tumor uptake of AuNPs through the osmotic retention effect and the coupling of tumor-specific targeting peptides^44^. Interestingly, AuNPs can contribute to tumor cell apoptosis by catalyzing the production of ROS, which disrupts the mitochondrial membrane potential and induces the release of apoptotic proteins, thereby triggering tumor cell death^45^. AuNPs are usually generated by microwave synthesis, laser ablation, uvirradiation, and chemical oxidation reactions. Adding antioxidants and stabilizers during the synthesis process improves the stability of AuNPs^46^. The high surface area-to-volume ratio of AuNPs provides the ability to load functional immunotherapeutic agents densely. Through covalent attachment to Au–S or Au–N molecules, therapeutic agents such as DNA, proteins, and antibodies are often immobilized on the surface of AuNPs, thus enhancing their anticancer ability. AuNPs can enhance tumor immune responses and be effective adjuvants for tumor vaccines^47^. AuNP size and shape can manipulate to stimulate various cytokine pathways. AuNPs can be designed to be around 20–100 nm, with small particles (<10 nm) being cleared by the kidneys and large particles (>200 nm) being cleared by the liver and spleen^48^. In addition, the abundant plasmon resonance on their surfaces confers high efficiency in absorbing and scattering light by the AuNPs, which makes them a good choice for combining with photothermal therapy and photodynamic therapy as tumor immunotherapy^49^. This intense light absorption capacity makes AuNPs exceptional contrast agents for photoacoustic imaging (PA), tumor-targeted radiography, and computed tomography/single photon emission computed tomography (CT/SPECT) imaging^50^. Despite the advantages described above, it is worth noting that the limited clearance of AuNPs by the body may result in the retention of excess AuNPs in the cell for a relatively long period, inducing toxic or carcinogenic lesions^48^.

#### 2.2.3 Iron oxide nanoparticles

Iron oxide nanoparticles (IONPs), which are mainly composed of iron (II, III) oxide (Fe_3_O_4_) and iron (III) oxide (Fe_2_O_3_), have been extensively studied for their potential as carriers for immunotherapeutic drug delivery^51^. IONPs can be prepared by multiple routes of chemical, physical, and biosynthesis^52^. Possessing attributes such as good biocompatibility and biodegradable properties, while the excess IONPs can be utilized and absorbed by various cells without triggering cytotoxicity^53^. Once in the physiological environment, IONPs are internalized by circulating monocytes, migrating toward tumor cells and differentiating into macrophages. A seminal study examined the propensity of IONPs to label TAMs in breast cancer. Their findings showed that TAMs preferentially phagocytose IONPs over malignant tumor cells, resulting in substantial tumor retention and sustained MRI contrast enhancement after intravenous injection^54^. The ability to monitor the distribution and migration patterns of immune cells *in vivo* using IONP-facilitated magnetic resonance imaging immune tracking provides an essential insight into the involvement of immune cells in the tumor treatment paradigm. This approach has been successfully used to track tumor-directed migration of cytotoxic T cells and organ-targeting behavior of natural killer cells^55^^,^^56^. The efficacy of IONPs in facilitating MRI-mediated immune tracking is closely related to the size of the IONPs, the surface charge density, and the magnitude of the applied magnetic field, and it has been demonstrated that IONPs with diameters of less than 10 nm are cleared by the kidneys. In contrast, IONPs with a diameter of greater than 200 nm have been shown to accumulate in the liver, thus underscoring the importance of rationally designing and adapting IONPs for this type of study^57^. The flexible surface properties of IONPs enable them to be effective loading platforms for a range of immunotherapeutic agents such as conventional drugs, cytokines, small interfering RNAs (siRNAs), adjuvants, immune vaccines, and immune checkpoint inhibitors, all of which can be delivered to the tumor cells to produce therapeutic effects^58^, ^59^, ^60^. IONPs can also be used in conjunction with photothermal therapy (PTT) and magnetothermal therapy (MHT) to induce more excellent antitumor responses^52^.

### 2.3 Bionic NPs

#### 2.3.1 Exosomes

Recently, exosomes derived from almost all cells and body fluids have also been developed as drug-delivery cells for tumor immunotherapy^61^. Biologically derived exosomes have the ability to deliver therapeutic cargo (nucleic acids, proteins, small molecule compounds) to recipient cells^62^. Unique biological origins confer excellent biocompatibility, low immunogenicity, low cytotoxicity, and biodegradability, making them promising drug delivery vehicles for tumor immunotherapy^63^. Therapeutic cargoes can be loaded into exosomes *via* endogenous (genetic engineering, transfection, transfection) or exogenous (electroporation, co-incubation) routes. genetic engineering, transfection) or exogenous (electroporation, sonication, co-incubation) routes to load into exosomes^64^. Currently, MSCs and dendritic cell sources have been used to treat various diseases in the clinic^65^. Studies have found that exosomes from plant and microbial sources can also be used as a drug delivery system in addition to extracellular milk^66^. The presence of a rich stroma and several immune-suppressing cells leads to an inferior outcome of conventional treatments for Pancreatic Ductal Adenocarcinoma (PDAC), such as chemotherapy, with a 5-year survival rate of <6%^67^. To improve this situation, a study by cheng et al.^68^ loaded galactoglucagon-9 siRNA into bone marrow mesenchymal loaded oxaliplatin (OXA) mesenchymal stromal stem cell (BM-MSC)-derived exosomes by electroporation to enhance immunotherapy and reprogramming of the TME. iEXO-OXA preferentially delivers to the tumor site and enhances the delivery of therapeutic agents under the protection of exosomal lipid bilayers. OXA significantly enhances PDAC therapy by down-regulating cytotoxic T-lymphocyte recruitment and Tregs, triggering anti-tumor immunity. Overall, exosomes are used as tumor immunotherapies to enhance immunotherapy and reprogram TME. In conclusion, exosomes have a promising future as a drug delivery platform for tumor immunotherapy. Still, exosome scale production, isolation and purification, and long-term storage are the bottlenecks limiting its development^69^.

#### 2.3.2 Neutrophils

Neutrophils make up 65%–80% of circulating leukocytes and are critical for clearing infections from inflammatory sites in the body^70^. Widespread presence in the circulation, phagocytosis, and the ability to cross physiological barriers make them candidates for achieving the goal of targeting therapeutic agents to tumor carriers^71^^,^^72^. Co-incubation experiments revealed that mesophilic granulocytes themselves can mediate the tumor-killing effect in a variety of ways, including stimulating stimulation of CD^4+^ and CD^8+^ maturation through the release of a variety of cytokines such as alerting, arginase-I, and MPO, or by increasing the influx of Ca^2+^ into the tumor^73^^,^^74^. Based on the ability of neutrophils to phagocytose nanoparticles, Zheng et al.^75^ conducte insert a fascinating study in which the antitumor drug adriamycin (Dox) was loaded onto magnetic mesoporous silica nanoparticles, which were subsequently conjugated with healthy mouse peripheral blood neutrophils, and then intravenously injected into a glioma model of an inflamed mouse, where neutrophils were recruited to the inflamed glioma site under the effect of chemoattractants or chemokine "pulling," and the release of the cargo of D-MMSNs was followed by the uptake of D-MMSNs through the infiltration of the glioma cells, resulting in a highly efficient anti-glioma.

#### 2.3.3 Macrophages

The tropism of macrophages or their predecessor monocytes for cancer-related cytokines (*e*.*g*., CSF-1, VEGF, TNF, IL-1, IL-5, etc.) and chemokines (*e*.*g*., CCL-5, 7, 8, 12, etc.) based on the ability of macrophages or their predecessor monocytes to cross physiological barriers make them another ideal vehicle for oncology drug delivery^76^. However, macrophages' innate foreign body scavenging effect to protect the body from bacteria and viruses makes free drug loading difficult. At the present stage, a "two-step" strategy is used to realize macrophage-based drug loading, in which therapeutic drugs are first loaded into conventional NPs and subsequently co-incubated with macrophages, utilizing the principle of antibody-antigen recognition or the macrophage "Trojan horse" effect to achieve drug loading in the macrophage membrane or lysate. Drug loading in macrophage membranes or lysates is achieved, followed by *in vivo* delivery of tumor-immune agents using macrophages as delivery vehicles^77^^,^^78^. For example, a study by Xie et al.^79^ developed a Dox-silica nanocapsule platform and subsequently internalized the therapeutic drug into the macrophage cytoplasm by co-incubation with murine macrophage cell line RAW264.7 cells, which was tested in the U87MG xenograft model and found to be effective in inhibiting tumor growth without causing systemic toxicity compared to macrophages using macrophage-loaded DOX alone. Good biocompatibility, deep tumor drug delivery, and good safety profile are the main advantages of macrophages as delivery vehicles, but low macrophage content in circulation and limited drug loading efficiency are the main obstacles limiting their development.

#### 2.3.4 Virus-like NPs

**Figure 2:**
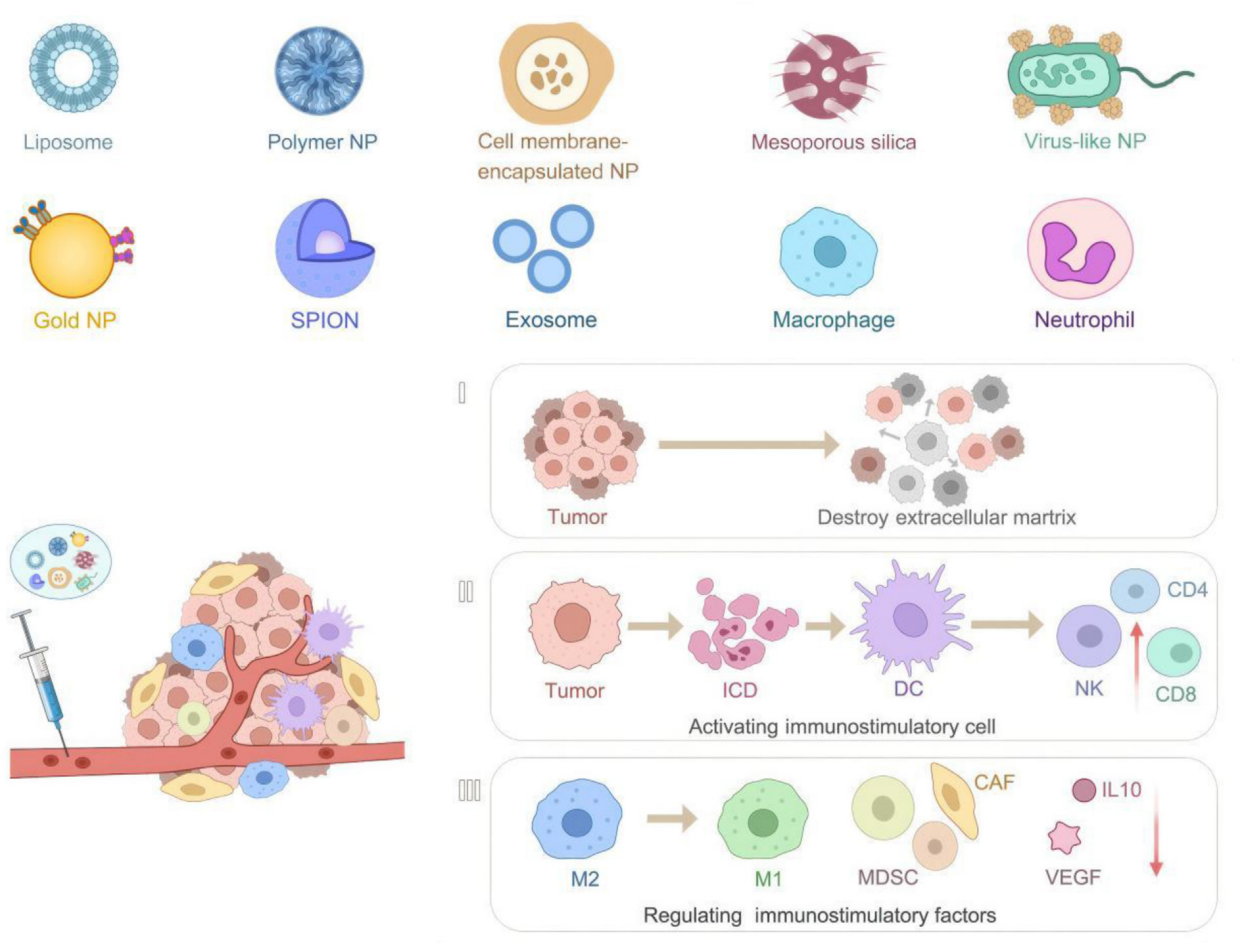
Strategies for the treatment of tumors with diverse NPs carrying therapeutic immunological agents.

Virus-like nanoparticles (VLPs) are nanoscale entities that mimic the structure of viruses but do not contain viral genetic components^80^. The ability of VLPs to elicit an immune response and the lack of risk of viral transmission makes them ideal vehicles for vaccine delivery. VLPs were initially used as vaccine vectors against hepatitis B, hepatitis E, and human papillomaviruses^81^^,^^82^. With the increasing understanding of viral structures and delivery vectors, cowpox viruses, adenoviruses, plant viruses that invade cowpea leaves, and retroviruses have been successfully developed as delivery vectors for tumor immunotherapy^83^ (as shown in Table 1^22^^,^^24^^,^^32^^,^^36^^,^^38^^,^^39^^,^^53^^,^^65^^,^^69^^,^^71^^,^^79^^,^^82^^,^^83^). VLPs extracted from cowpea leaf phytoplasma viruses were first purified by protein hydrolysis to L and S capsid proteins and then further purified by volumetric exclusion chromatography, immobilized metal ion chromatography, and size exclusion. Nucleic acids, proteins, and chemotherapeutic drugs can be encapsulated in VLPs by covalent and non-covalent techniques^84^. Non-covalent approaches can eliminate the need for additional cargo modification, whereas covalent approaches ensure effective encapsulation and retention of the payload. To fully utilize the therapeutic effect of loaded drug VLPs, circumventing the attack on the body's immune system is crucial. Surface modification of VLPs using PEG/CD47/cell membranes can confer a "stealth" capability and minimize their removal by the organism's immune system^85^. For example, an investigation found that empty cowpea mosaic virus (CMV) VLPs without a nucleic acid capsid were detected by toll-like receptors 2, 4, and 7. This recognition recruits and activates DCs, suppresses regulatory T cells, and is an effective *in situ* cancer vaccine when administered intratumorally. 2023 A study conducted demonstrated that intratumorally injected VLPs delivering cyclic guanosine adenosine monophosphate (cGAMP) induced differentiation of circulating tumor-specific T cells, reduced regulatory T cells, and, in combination with PD1 blockade to produce a synergistic antitumor response. This evidence supports combining VLPs with immunotherapy as a viable therapeutic strategy in tumor therapy^86^ (Fig. 2).

**Table 1:** Advantages and disadvantages of various types of NPs as delivery vectors for Tumor immunotherapy.

Nanoparticle type	FDA-approved for human	Advantage	Disadvantage	Ref.
Organic NPs				
Liposome	First FDA-approved human, liposome-encapsulated Doxil, DaunoXome already in clinical studies	Biocompatible, easily artificially modified, biodegradable, high drug-carrying capacity	Thermal instability, quick and rapid removal of the body, high production costs	22,24
Polymers	Multiple polypropylene glycolide-co-glycolic acid approved by FDA for drug delivery applications, polymer Abraxane in clinical use in 2005	Biodegradable, high drug-carrying capacity, can be artificially modified	Structural heterogeneity, particle instability, slow drug release, potential immunogenicity, large size, higher production cost	32,36
Inorganic NPs				
Mesoporous silica	N/A	Low toxicity, low cost, artificially modified, biodegradable	Short half-life, requires surface modification for targetability	38,39
Au NPs	Small number of AuNPs approved for therapeutic drug delivery applications	Good optical properties for deep tissue imaging and treatment	Non-specific, toxic or carcinogenic lesions induced by prolonged intracellular storage	
IPONs	Multiple IPONs approved by FDA for cancer thermotherapy applications.	Good biocompatibility, can be used as a source of iron, can be manipulated by magnetic fields, biodegradability	May cause oxidative damage, low water solubility	53
Biomimetic NPs				
Exosome	Currently only exosomes of MSC and dendritic cell origin are used in clinical studies	Highly biocompatible, essentially non-immunogenic, easy to artificially modify, diverse means of drug loading, wide range of sources	Difficulty in large-scale production, lack of standardized separation and purification means, not easy to store for a long time.	65
Neutrophil	N/A	Abundant in blood, tumorigenic, crosses physiological barriers	Inefficient drug loading	69,71
Macrophage	N/A	Convergent, crosses physiological barriers	Low blood levels and extremely inefficient drug loading	79
Virus-like NPs	Adenovirus, Modified vaccinia virus Ankara	Lack of natural genome is not infectious and can be considered modified with drug loading capacity	Immunogenicity, poor stability, risk of inducing cancer	82,83

NP_S_, nanoparticles; FDA, U.S. food and drug administration; IPONs, iron oxide nanoparticles; MSC, mesenchymal stem cell; N/A, not applicable.

## 3 Transcending histological barriers: Role of nanomaterials in cancer immunotherapy

Specifically, in the biomedical field, nanomaterials are used to develop drug delivery systems to enhance the pharmacokinetics and pharmacodynamics of therapeutic drugs to improve their bioavailability and mitigate harmful side effects. The paradigm shift from analyzing tumor cells in isolation to evaluating entire tumor tissues and organs is an essential milestone in oncology research. It has driven the rapid development of cancer immunotherapy. However, cancer immunotherapy delivery systems suffer from numerous drawbacks, mainly due to physiological and pathological barriers preventing effective tumor cells from targeting ^87^. Therapeutic agents encounter myriad biological and pathological barriers in targeting tumor sites. These barriers include the endothelial barrier, the blood–brain barrier (BBB, especially in the case of neurologically related diseases), the barrier formed by the tumor tissue itself, and the metastatic barrier between cancer cells. This intricate situation results in only a small percentage (approximately 0.7%) of administered drugs effectively reaching the cancer cells and exerting therapeutic effects^87^. However, NPs have attracted much attention for their remarkable adaptability. Their outstanding chemical versatility, excellent biocompatibility, ease of artificial modification, and ample drug-loading capacity have pushed them to the forefront of scientific research. Next, we will discuss the various barrier systems NPs face after blood entry and strategies to overcome them.

### 3.1 Blood barrier

The reticuloendothelial system's (RES) innate ability to scavenge foreign substances is essential for maintaining homeostasis. Yet, this defense mechanism of the body is a challenge for the adequate circulation of therapeutic NPs in the blood system^88^. Upon entry into the bloodstream, a variety of conditioning proteins (*e*.*g*., immunoglobulins, components of the complement system (*e*.*g*., C3, C4, and C5), serum proteins, etc.) in the bloodstream can come into contact with the injected NPs through Brownian motion, and then bind to the surface of the NPs, with the help of electrostatic, ionic, and hydrophobic/hydrophilic forces leading to formation of protein corona, which stimulates phagocytosis to attach to and endocytose the bloodstream nanoparticles, and subsequently, the formation of a corona. Secretory enzymes and other oxidatively reactive chemokines break down the endocytosed substances, significantly reducing circulating drug concentrations^89^^,^^90^. Prolonging therapeutic drug circulation time, to some extent, has been reported to increase drug accumulation at the target site^91^. NPs can overcome blood vouchers through the following strategies.

#### 3.1.1 Adjust the physicochemical properties

**Figure 3:**
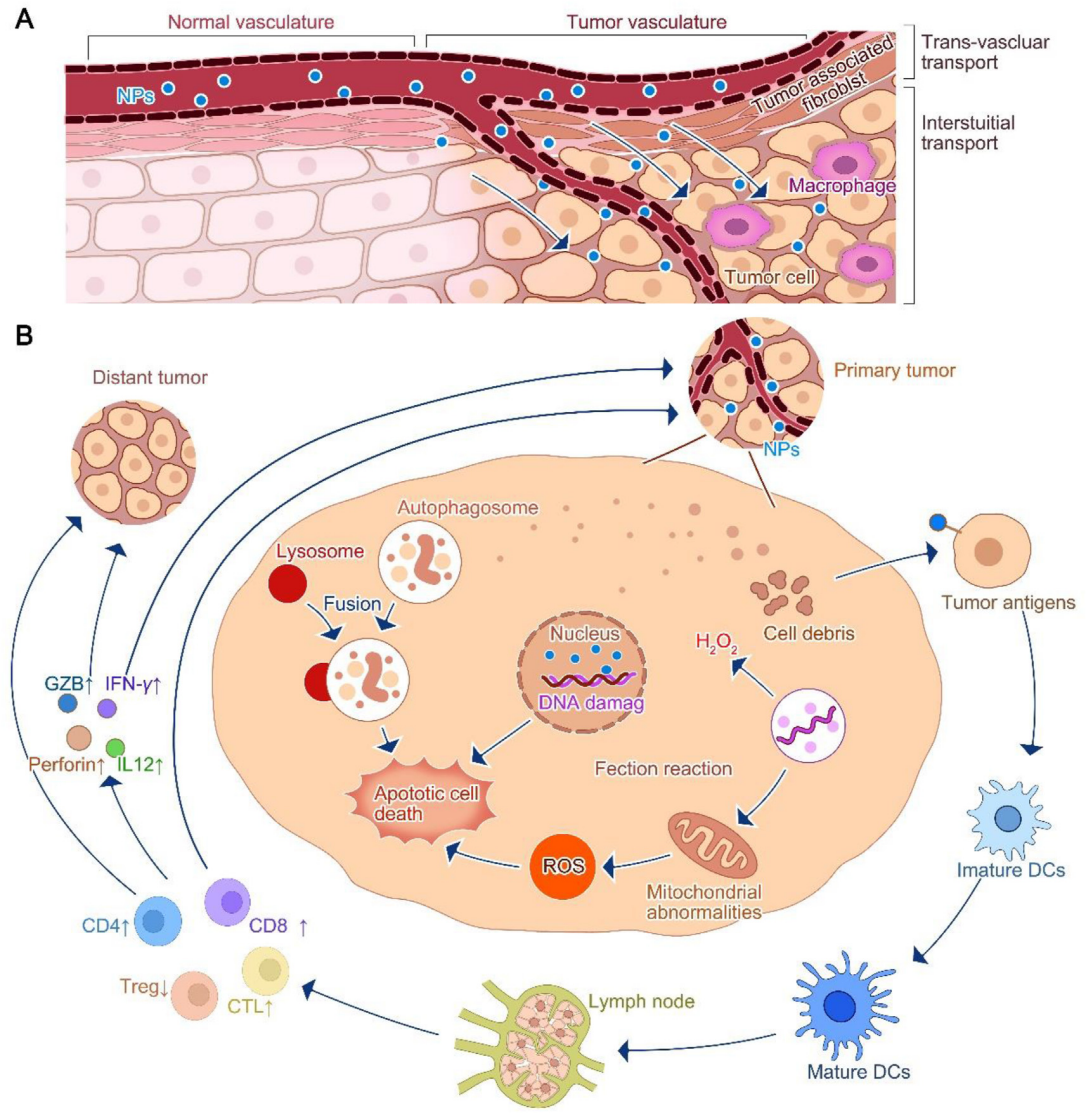
Strategies for the therapeutic role of NPs carrying therapeutic cargoes in the circulation. (A) The mode of transport of NPs in normal blood vessels as well as tumor vessels, in tumor vessels by enhancing the retention effect from extravasation to the tumor microenvironment, targeting tumor cell immune-related drugs will limit tumor development by destroying the tumor stroma and remodeling the tumor microenvironment. (B) NPs entering tumor cells can induce apoptosis by breaking the DNA of tumor cell nuclei, increasing the production of reactive oxygen species and autophagy, etc. Apoptotic tumor cell lysates stimulate the activation of DCs cells in the form of antigens. Activated DCs stimulate lymph nodes to produce various effector T cells (*e*.*g*., CD8T, CD4, CTL, etc.) to kill tumor cells directly, while on the other hand, these effector T cells further limit tumor development by secreting various cytokines (*e*.*g*., IL-12, IFN-*γ*, etc.) and GZB.

Widely accepted consensus is that the physicochemical properties of NPs (*e*.*g*., size, shape, and charge) affect the ability of the body's blood system to clear them; as mentioned earlier, after entering the blood, NPs smaller than 10 nm, the kidneys quickly clear, and NPs larger than 200 nm are quickly cleared by the liver and spleen, comprehensive assessment and design of NPs particle size range of 10–200 nm can effectively circumvent the blood system to remove them^91^. Upon entry into the bloodstream, compared to neutral or negatively charged serum proteins, there is a stronger tendency for positively charged polymeric NPs, the formation of the protein corona, and easy clearance by the reticuloendothelial system^92^. In addition, NP curvature and aspect ratios correlate with macrophage internalization, and filamentous or worm-like NPs are more effective in circumventing macrophage phagocytosis than ellipsoidal, cylindrical, and discoidal NPs. Therefore, the rational design of NPs contributes to prolonged blood circulation^93^ (Fig. 3A).

#### 3.1.2 Surface modification

One possible approach is to coat NPs with an inert polymer coating to cloak them invisibly. In the field of drug delivery, PEG has been extensively studied as an "invisible" polymer^94^. PEG grafted on the surface of NPs effectively shields proteins and other biomolecules from surface adsorption by forming a spatially hydrated "cloud" layer, rendering the NPs invisible to the MPS and thus prolonging blood circulation time^95^. For example, one study developed amphoteric cyclodextrin NPs containing PEG-modified quercetin (QTN) and the ginsenoside Rg3, which induces endoplasmic reticulum stress and autophagy in tumor cells, leading to immune-causing tumor cell death, including through the upregulation of phosphorylation of IRE1 and PERK proteins. This effect is further augmented by ROS generated by QTN. Evaluation of the drug half-lives of the free and PEG-encapsulated co-formulated drugs in a CRC mouse model revealed that the lowest levels of the free drug were detected at 8 h post-injection (20 min for *t*_1/2_ QTN and 10 min for Rg3). In contrast, the targeted co-formulated drug was cleared from the plasma at a significantly slower rate (1.4 h for *t*_1/2_ QTN and 1.3 h for Rg3), which suggests that the spatial modification of PEGs prevents NPs from being modified in the endoplasmic reticulum and autophagy (Fig. 3B). Spatial modification prevents the nonspecific uptake of serum proteins by NPs and improves the circulation of NPs^96^.Another strategy involves surface modification of the nanoparticles using CD47 or a peptide derivative of this marker. This alteration reduces the uptake of nanoparticles by phagocytes by sending a "do not eat me" signal to the organism^97^. IIn addition, NPs are encapsulated by biofilms or cell-derived vesicles of erythrocytes, leukocytes, macrophages, platelets, etc^98^. This process involves the mechanical removal of surface cell membranes from immune cells, erythrocytes, stem cells, cancer cells, etc., using ultrasound, freeze-thaw, hypotonic lysis buffer, and extrusion. These shed cell membranes are then wrapped onto the surface of core NPs using microfluidic electroporation, ultrasound/extrusion, and extrusion/electroporation^99^^,^^100^. These membrane-encapsulated nanoparticles, known as "self-friends," carry "self-recognition molecules" and "self-labeling molecules" that allow the nanoparticles to evade clearance by the immune system, as well as to have extremely high biocompatibility and prolonged blood circulation properties^98^. A study found that leukocyte membrane-encapsulated nanoporous silica particles were protected from the modulatory effects of high-abundance serum proteins, which resulted in a 75% reduction in nanoparticle uptake by mouse phagocytes and an approximately 1% reduction in nanoparticle uptake by human THP-50 phagocytes, prolonging the circulation time of the particles and improving tumor accumulation^101^.

### 3.2 BBB

**Figure 4:**
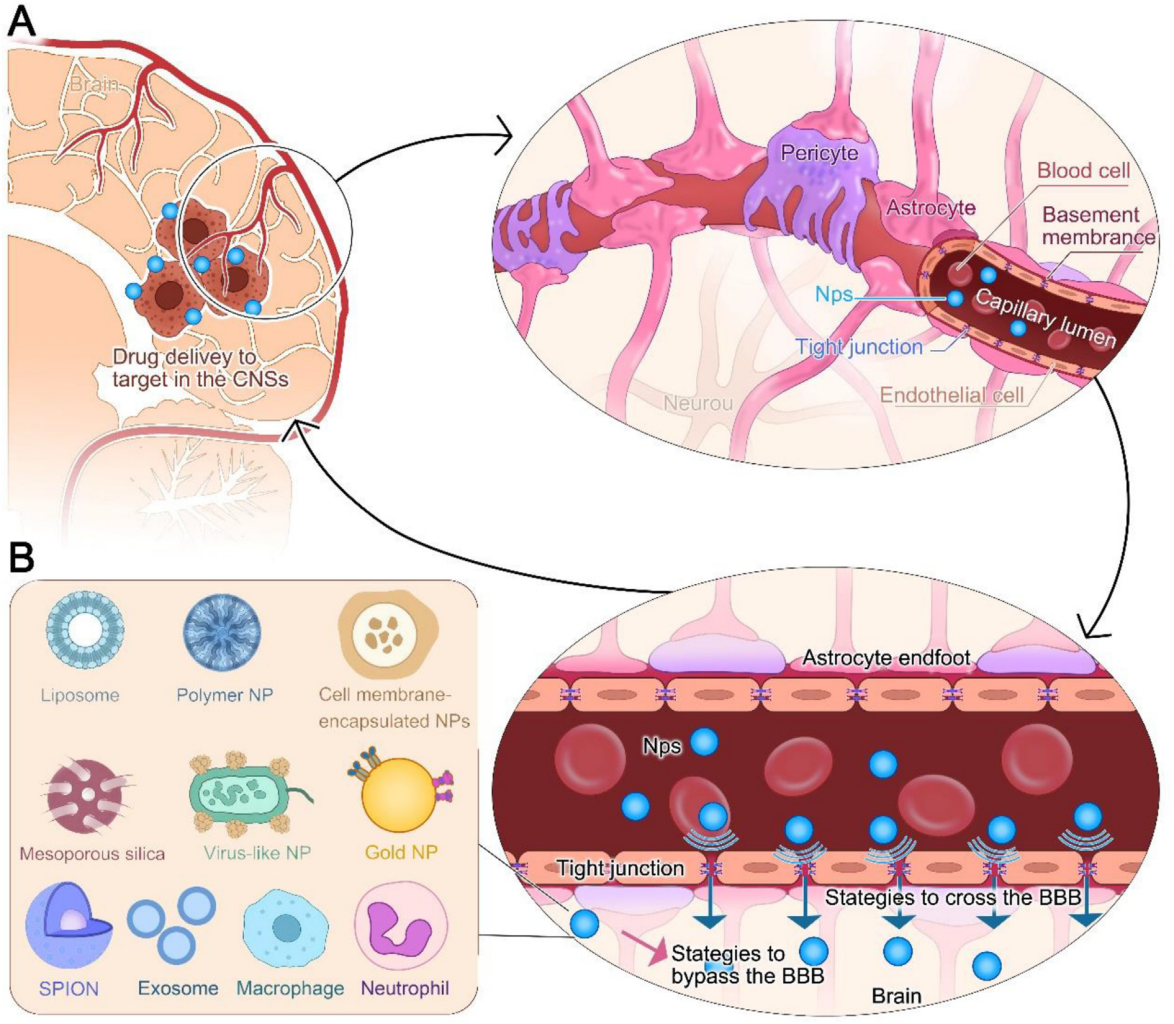
The structure of the blood‒brain barrier (BBB) and strategies for NPs to traverse BBB. (A) BBB consists mainly of endothelial cells tightly connected between successive cells and surrounded by a basal glycoprotein layer shared with pericytes and astrocyte ends; it maintains the central nervous system in a relatively closed state, limiting the entry of many substances to protect the central nervous system from pathogens and bacterial toxicity. (B) Diverse NPs loaded with immune-related drugs will cross the BBB through ligand-receptor-mediated, carrier-mediated, and adsorption-mediated modifications to target therapeutic drugs to tumor cells for therapeutic effects.

The BBB is a semi-permeable boundary consisting of tightly packed endothelial cells of the nerve parenchyma surrounded by a basal glycoprotein layer shared with pericytes and astrocyte end-feet. This structure isolates the central nervous system (CNS) from peripheral blood circulation. It facilitates the immediate delivery of oxygen and nutrients based on neuronal needs, tightly controls the influx of other molecules and particles into the CNS, and establishes a relatively stable environment within the BBB that protects the CNS from invasion by pathogens and toxins^102^ (Fig. 4A). However, the most effective intravenous formulations have been reported to deliver 5% of the initial dose to the brain when a CNS lesion is present^103^. The BBB's limitation on drug delivery, not the lack of drug candidates, makes the effective treatment of many CNS disorders (*e*.*g*., glioblastoma, Parkinson's disease, cerebral infarction, etc.) challenging^104^. Notably, when modified, nanoparticles enable BBB penetration and subsequent drug delivery to target cells. Specific ligands are explicitly coupled to NPs to increase their recognition by the BBB, while NPs cross the BBB by migration. NPs can overcome the BBB through the following strategies.

#### 3.2.1 Recognition of receptors on the BBB

Receptors for transferrin, insulin, insulin-like growth factor, or lipids expressed on the luminal surface of brain endothelial cells (BECs) are essential for the delivery of nutrients required to maintain the regular physiological activity of the brain^105^. It has been reported that coupling Angiopep-2, RVG-29, and polysorbate 80 to therapeutic drug-carrying NPs is a current strategy to cross the BBB by hijacking the corresponding BEC receptors. After blood entry, adequate blood circulation disperses the drug-carrying NPs into Hijacking the corresponding BEC receptor, which is currently a feasible strategy to cross the BBB^91^. After entry into the bloodstream, adequate blood circulation disperses the drug-carrying NPs in the official cavity of the blood–brain barrier. The specific ligands bind to the receptor and change the conformation, triggering receptor-mediated transmigration (RMT) to deliver the drug load effectively. For example, a study Han et al.^106^ loaded adriamycin onto polymeric NPs modified with Angiopep-2. After *in vivo* injection, Angiopep-2 drives transcytosis through BBB and triggers endocytosis of brain metastatic targets by specifically binding to LDL receptor-related protein 1 (LRP1), which is highly expressed in brain metastatic carcinoma endothelial cells. It was found that the endocytosis of A-NPs/DOX-treated cells was 3.39-fold and 3.93-fold more significant than that of free DOX and NPs/DOX, respectively. DOX content in DOX-treated cells was 3.39-fold and 1.93-fold higher than free DOX and NPs/DOX, respectively. Similarly, a study developed a disulfide-bonded crosslinked polymer NP design loaded with the angiopoietin-2 peptide and Cas9/sgPLK1 affixed with a dual-acting ligand. In tumor cells containing high concentrations of glutathione (GSH), the NPs protect the cargo from ribonuclease (RNase) degradation. Upon release, angiopoietin-2 peptide binds to low-density lipoprotein receptor-related protein-1 (LRP-1), which is present in high concentrations in glioblastoma (GBM), thereby enhancing BBB permeability and GBM targeting *via* receptor-mediated transcytosis. Anti-Polo-like kinase 1 (PLK1) Cas9/sgPLK1 inhibited GBM cell proliferation and triggered apoptosis by markedly suppressing the cellular mitogenic protein PLK1 expression within the GBM. Remarkably, these NPs reached a peak drug concentration of 8.4% at 11 h post-injection, which greatly exceeds the typical accumulation of only 1%–5% in the brain by other delivery systems^107^^,^^108^. In summary, the presence of the BBB promotes CNS disease progression but also provides a target for therapy.

#### 3.2.2 Recognition of transporter proteins on the BBB

Recognition of carrier proteins on the BBB: small molecules such as glucose, amino acids, nucleosides, and monocarboxylic acids are transported bi-directionally across the BBB *via* carrier protein-mediated transporter (CMT)^91^. A variety of carrier proteins [*e*.*g*., sodium-coupled glucose transporter (SGLT), monocarboxylic lactate transporter 1 (MCT1), cationic amino acid transporter 1 (CAT1), and l-type amino acid transporter 1 (LAT1)] have been reported to be involved in the transport of small molecules across the BBB^109^. At this stage, a promising strategy modifies NPs carrying therapeutic immune pharmaceuticals with a mimetic transporter protein substrate, which mimics the specific binding of the substrate to carrier proteins to drive translocation and thereby enable medicinal drugs to break through the BBB for transport (Fig. 4B). Based on previous findings that liposomes modified with aminophenyl-*α*-d-mannopyranoside (MAN-LIP) facilitated penetration into the brain *via* the BBB and also targeted selected intracerebral regions, including the cortex, cerebellum, brainstem, hippocampus, and pontine nuclei, a continuity experiment by comparing the results of liposome-alone versus MAN-LIP brain delivery, and DiR imaging found that MAN-LIP had much stronger brain signals than LIP alone mice, and phenobarbital inhibition confirmed that MAN-LIP enhances BBB and tumor cell delivery by binding specifically to GLUT1 and GLUT3 in the blood–brain barrier and glial cells^110^^,^^111^.

### 3.3 The tumor barrier

The "seed and soil" hypothesis of tumor progression vividly illustrates the intricate interplay between tumor cell evolution and its microenvironment. Here, the "seed" represents the tumor cell, while the "soil" comprises endothelial cells, fibroblasts, immune cells, various stromal cells, and constituents of the extracellular matrix (ECM)^112^. The TME is distinguished by suboptimal blood supply, hypoxia, acidosis, and interstitial hypertension^113^. The primary challenge in achieving therapeutic drug efficacy is surpassing the tumor barrier, encompassing the tumor vascular barrier and stromal barriers^114^. The hypoxic microenvironment disrupts the dynamic equilibrium between pro-angiogenic molecules and anti-angiogenic factors within the tumor, resulting in numerous abnormal structures, inadequate normal branching, and hypofunctional endothelial cells, impeding successful drug delivery to tumor cells. The tumor-stromal barrier primarily consists of a gelatinous, dense ECM, elevated interstitial fluid pressure (IFP) induced by inefficient vascular and lymphatic drainage, and cancer-associated fibroblast (CAF) stromal cells. NPs can overcome the tumor barrier through the following strategies.

#### 3.3.1 In passive targeting

**Figure 5:**
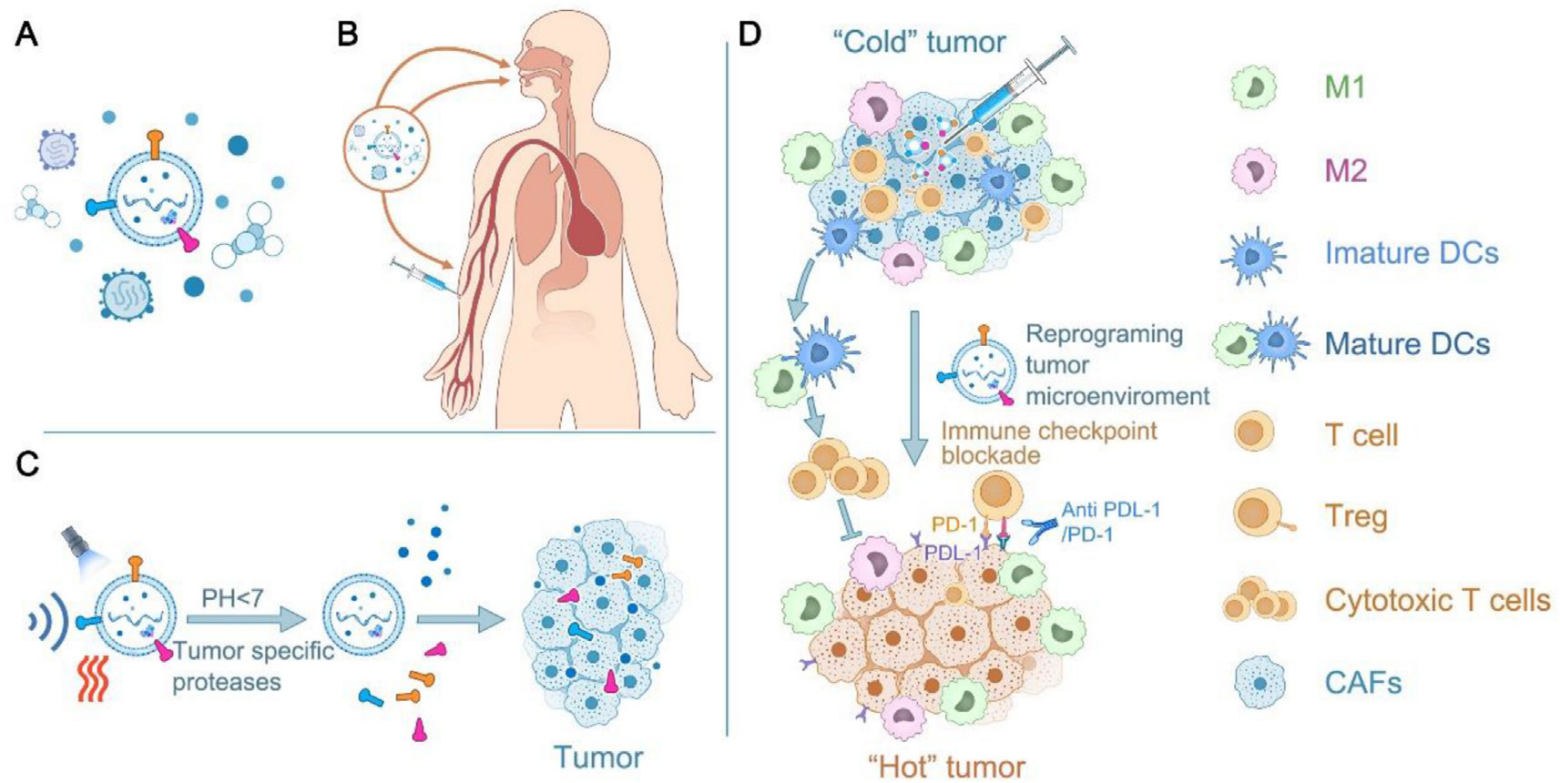
NPs combined with tumor immunotherapy strategies. (A) Diverse drug-carrying NPs. (B) Strategies for nanoparticle entry into the organism. (C) NPs that enter the body can induce immune drugs from NPs in response to specific stimuli (*e*.*g*., remote light, heat or multiple enzymes or acidic conditions in the tumor microenvironment). (D) These drugs can reverse the suppressive tumor microenvironment by increasing M1, cytotoxic T cells, or decreasing immunosuppressive cells, M2, Treg, etc., changing a “cold tumour” into a “hot tumor”.

NPs can enhance drug accumulation in the tumor *via* EPR effects. Specifically, NPs smaller than 200 nm exploit the fenestration capability of the tumor vascular system and the absence of a fully functional lymphatic drainage system in tumor cells to amplify intra-tumor drug concentration and NP retention time (Fig. 5A and B) Ultrasound, radiation, high temperature, and nitric oxide (NO) treatments can further enhance tumor vascular permeability, augmenting NPs’ EPR effect. For example, in one study of NO-NPs encapsulated by DOX, high concentrations of glutathione (GSH) in the TME destabilized the structure of NPs, and the released NO dilated tumor blood vessels and enhanced vascular permeability, enhancing the therapeutic effect of DOX into tumor cells in the form of positive feedback^115^ (Fig. 5C). An additional strategy to strengthen the breach of the tumor vascular barrier by NPs is the rational modification of NPs to augment their binding to tumor endothelial cells and amplify their therapeutic impact. For example, one study coupled CGKRK (Cys-Glys-Lys-Arg-Lys) peptide and tumor penetrating peptide iRGD (sequence CRGDKGPDC) into ferrous oxide nanoparticles for successful application in the treatment of GBM. CGKRK peptide provides a specific tumor vascular targeting function to transport NP to tumor vascular cells and into mitochondria to kill tumor cells through the dramatic toxic effect of CGKRK; at the same time, applying iRGD further enhances nanoparticle penetration into extravascular tumors^116^.

#### 3.3.2 Proactive targeting strategy

The tumor matrix barrier for NP drug delivery can be alleviated by loading NPs with biochemical enzymes like collagenase and hyaluronidase, which degrade the ECM and enhance drug diffusion within the tumor barrier^117^. Traditional methods such as ultrasound, photothermal, and thermal therapies, as well as biochemical enzymes, have been shown to enhance tumor immunotherapy by destroying the ECM. However, the non-negligible damage to normal tissues leads to significant limitations in coordinated immunotherapy by these means^118^^,^^119^. Based on the inherent advantages of NPs, Zhou et al.^120^ tethered adriamycin and hyaluronidase PH20 (rHuPH20) onto poly (lactic-co-glycolic acid) NPs modified with PEG. PEG modification reduced NP degradation by the endothelial barrier. rHuPH20 was anchored to the ECM and hydrolyzed hyaluronic acid, a significant component of the ECM, thereby reducing IFP. These modified NPs effectively inhibited the growth of aggressive 4T1 tumors at minimal doses. In addition, a guided article found that in stromal vascular tumors, regardless of NP size or presence of targeting ligands, NPs are initially preferentially distributed to tumor-associated fibroblasts, overcoming tumor-specific delivery barriers and coordinating the inhibitory TME by utilizing CAF as an *in situ* cytokine or cytotoxic protein production reservoir to improve antitumor outcomes in the current margins of stromal vascular tumors^121^. In addition, the design of small-size NPs helps to overcome the stromal barrier and target tumor cells. A study evaluating the effect of combining PDT with positively charged size 20 and 180 nm for tumor immunotherapy found that a high tumor accumulation efficiency of 6.94% was achieved in the 20 nm size group (about 1.8 times higher in the 180 nm group)^122^.

Furthermore, the "smart" design of TME-responsive NPs triggers the controlled exposure of targeting ligands upon drug release, enhancing drug tumor targeting^123^. Rapid proliferation of tumor cells leads to significant oxygen depletion within the TME, establishing a prominent hypoxic zone within the tumor. Hypoxia-responsive NPs have been designed to exploit this characteristic. For example, NPs loaded with azo moieties, human serum albumin (HSA), and oxaliplatin (HCHOA) were developed by zhao et al.^124^ Under tumor hypoxia, the azo moieties were cleaved and reassembled into therapeutically active ultra-small compounds, significantly enhancing their intra-tumor penetration. The pH-responsive nature of NPs is often engineered by incorporating certain ionizable weak acidic groups (proton donors) or weak primary groups (proton acceptors) into the NP matrix. These ionizable groups remain stable under physiological conditions characterized by a neutral pH (7.4). However, upon exposure to the more acidic microenvironment of solid tumors (lower pH), these groups undergo hydrolytic reactions. This pH transition enhances the cellular uptake of the drug payload, facilitated by surface ionization and electrostatic interaction with the cellular membrane^125^. Rapid tumor cell proliferation, metastasis, and invasive behavior are intrinsically linked to the overexpression of certain enzymes within the TME, such as matrix metalloproteinases (MMPs), hyaluronidase, legumain, and NAD(P)H: quinone oxidoreductase-1 (NQO1). The dysregulated expression of these enzymes can serve as stimuli for endogenous tumor targeting. For instance, PEG-DOX conjugated micelles, modified with a tetrapeptide linkage comprised of alanine-alanine-asparagine-leucine, are internalized by tumor cells *via* endocytosis. High concentrations of legumain proteins in the TME cleave the tetrapeptide bond, releasing free DOX. Consequently, DOX can infiltrate the nucleus and cytoplasm of tumor cells, where it exerts its cytotoxic effects^126^.

## 4 Exploring the synergistic potential of nanoparticles in cancer immunotherapy

### 4.1 NPs-conjugated cancer vaccines

Vaccines elicit innate and adaptive immunity within the host organism^127^. Traditional cancer vaccines primarily comprise antigens that instigate the immune response and adjuvants that amplify this response when co-administered with antigens. Clinically employed adjuvants include aluminum hydroxide, phosphate, and Pattern Recognition Receptor (PRR) ligands^128^. However, the suppressive immune microenvironment, low antigen immunogenicity, and inadequate targeting render conventional tumor vaccines relatively ineffective in immunotherapy^129^. NPs emerge as a potential solution to these issues, offering advantages like precise targeting, facile surface modification, and biocompatibility.

In the context of cancer vaccine research, NPs could enhance immunotherapy through the following means: (1) NPs could act as adjuvants themselves, bolstering immunotherapeutic efficacy. The electrostatic co-assembly of designed copolymers with iron oxide nanoparticles and STING agonist MSA-2 into a library of acid-ionized iron nano adjuvants has shown significant inhibitory effects on established tumor growth, effectively improved lymphatic delivery of tumor antigens and offered long-term antitumor immunological memory effects^130^. (2) NPs could carry multiple antigens/adjuvants to elicit complex immune responses. They can simultaneously accommodate and co-deliver different antigens/adjuvants, which could be loaded into NPs or attached to the NP surface. One investigation utilized a dual antigen encapsulated into a spherical nucleic acid (SNA) nano platform, demonstrating a substantial reduction in mouse lymphoma tumor progression and an increase in memory T cell levels^131^. (3) NPs can act as precise drug delivery vehicles for tumor vaccines, targeting specific cells. Conventional tumor vaccines, when administered intravenously or intramuscularly, primarily accumulate in the liver and injection site, often causing liver damage in animal models. Low dosage targeting results in poor tumor vaccine efficacy; NPs can remedy this issue with their engineered precision targeting. For instance, macrophage-encapsulated polymeric SN38 lipid nanoparticles (mSLP) demonstrated an enhanced ability for immune evasion, prolonged blood circulation, and superior homing effect. In a 4T1 breast cancer mouse model, mSLP outperformed unencapsulated vaccines to inhibit primary tumor growth and reduce metastatic organ lesions^132^.

TNPs can act as precise drug delivery vehicles for tumor vaccines; these findings suggest that the future design of composite particle tumor vaccines incorporating antigens, adjuvants, and NPs might prove more effective for tumor immunotherapy, promising a more significant therapeutic outcome.

### 4.2 NPs combined with immune checkpoint inhibitors

Immunotherapies focusing on programmed death 1 (PD-1) interaction with its ligands, PD-L1 and PD-L2, have heralded a novel epoch in oncology^133^. Antibodies that obstruct immune checkpoints, namely Cytotoxic T-Lymphocyte Associated Protein 4 (CTLA-4) and PD-1/PD-L1, have proven highly successful in cancer immunotherapy. Current FDA-approved immune checkpoint inhibitors (ICIs) include nivolumab, nivolumab, ipilimumab, and tremelimumab^134^. However, their further development is curtailed by factors such as the low immunogenicity of tumors, suppressive immune microenvironment, and patient tolerance^135^. NPs can optimize the efficacy of ICis in tumor immunotherapy by altering their biodistribution and modulating endogenous and exogenous factors during treatment. Specifically, NP delivery systems enhance the therapeutic effect by carrying ICIs or gene editing systems to remodel TME, enhance the body's anti-tumor immunity, control the frequency of drug release, and reduce collateral damage to normal tissues.

1. NPs can deliver one or more immune checkpoint antibodies/drugs to augment the immune checkpoint blockade response. Conventional ICB treatment suffers from lower tumor-infiltrating effector T cells and a lack of tumor neoantigen load^136^. However, applying ICIs against PD-L1 and PD-1 has shown reduced mortality in advanced small-cell lung cancer. Co-delivery of low-dose PLK1 inhibitor (volasertib) and PD-L1 antibody by NPs, for instance, has been reported to upregulate PD-L1 *via* the MAPK pathway, enhancing the ratio of CD8^+^ lymphocytes to regulatory T cells (Tregs), and remodeling the suppressive TME to an immune-permissive state^137^ (Fig. 5D). While Gao et al.^138^ Developed loading PD-1 signaling pathway peptide inhibitor (AUNP-12) and indoleamine-2,3-dioxygenase (IDO) inhibitor tumor cascade targeting the NLG919@Lip-pep1 system, after blood entry, AUNP-12 for the tumor cells high expression of PD-L1 significant targeting to achieve the delivery system in the tumor accumulation, and subsequently in the matrix metalloproteinase-2 (MMP-2) cleavage triggered the dissociation of AUNP-12, which restored T cell activity by precisely blocking the PD-1 signaling pathway. *In vivo* distribution studies revealed that DiD@Lip-PEP1 preferentially targeted tumor cells while DiD@Lip-PEP1 was preferentially distributed to the liver, confirming that AUNP-12 coupled to enhance tumor targeting. This result fully testifies that it is feasible at this stage to design NPs to display specific ligands on their surfaces against receptors intrinsic or overexpressed in the tumor target region and utilize the ligand-receptor clear recognition principle to enhance the particular targeting delivery strategy of therapeutic drugs.
2. NPs can modulate the TME. Co-encapsulating CD47 antibody and STING agonist into liposomal NPs with stealth function has been demonstrated to enhance phagocytosis synergistically by altering microglia and macrophages' phenotypes, reversing immune suppression and converting “cold” to “hot” tumors^139^.
3. Besides carrying ICB-related inhibitors, NPs can deliver targeted gene editing systems associated with ICBs^140^; for instance, the CRISPR/Cas9 systems play a vital role in tumor immunotherapy by permanently suppressing genes and selectively knocking down immune checkpoint genes^141^.
4. NPs can control the frequency of drug release. The typical dosing protocol involves administering multiple immune checkpoint drugs at two or three-week intervals to maintain their efficacy. Some studies have demonstrated that solid LNP can extend the therapeutic effect by inhibiting the rate of siRNA release^142^^,^^143^.

The combination of ICBs and NPs-mediated therapies can potentially enhance tumor immunotherapy. For example, one study encapsulated chemo platin complexes with pH-responsive nanoparticles and found that combining this approach with ICB treatment could activate the STING pathway^144^^,^^145^.

In conclusion, NPs hold considerable potential to reshape the future of ICBs in cancer immunotherapy by overcoming the inherent limitations of these treatments.

### 4.3 NPs and monoclonal antibodies

The advent of rituximab as a therapeutic solution for B-cell non-Hodgkin's leukemia, approved by the FDA in 1997, catalyzed the robust growth of monoclonal antibodies (mAbs) for tumor immunotherapy^146^. This field has seen considerable successes over the last few decades, owing to the capability of mAbs to inhibit tumoral activity and promote their eradication through immune enhancement mechanisms^147^. A plethora of mAbs, such as trastuzumab, cetuximab, and ofatumumab, among others, have been crafted and employed in the fight against cancer cells^148^. However, their clinical application has been plagued with setbacks such as inadequate tumor penetration, extensive side effects (including fever, headaches, and muscle pains), off-target effects, and other deficiencies, thereby stalling the progression of mAb application in tumor immunotherapy. Recent evidence points to the promise of NPs as competent delivery vehicles designed for mAb loading and protection. Such a configuration can attenuate their degradation, boost their activity, and offer precise mAb release^149^.

Currently, strategies to functionalize nanomaterials through coupling techniques are gaining attention. For instance, a study aimed at integrating the epidermal growth factor receptor cetuximab (Ctxb) into zeolite nanocrystals found that based on accelerated high-speed uptake by tumor target cells, it could achieve therapeutic effects without compromising cellular activity. It suggested that combining monoclonal antibodies and nanoengineering could enhance tumor treatment^150^.

NPs can augment the therapeutic effect on tumors through the following strategies: as carriers, they deliver high concentrations of one or more antibodies, thus improving antibody-antigen binding and amplifying tumor immunotherapy efficacy. NPs, being several orders of magnitude larger than small therapeutic molecules, can be designed to carry a high density of one or more antibodies on their surface or within their core^151^. A study by Tao et al.^152^ Compared the therapeutic effect of Herceptin coupled with functionalized gold nanoparticles (AuNPs) against Her2-positive cancer with free antibody molecules. The results demonstrated that nano-coupled drugs significantly influenced the binding kinetics compared to free antibody molecules. Notably, the team observed that increasing the molar ratio of Herceptin to AuNP from 25 to 150 transitioned the antibody-antigen binding from a monovalent to a bivalent model, further enhancing the immunotherapeutic effect on tumors. EPR effects have been associated with PEG-modified NPs for long circulation and improved tumor targeting. A study that designed nanoparticles of driamycin DI17E6 (a monoclonal antibody against *α*_*v*_*β*_*3*_ integrin) modified with PEG found that, compared to the free drug, the complex nano-drug exhibited stronger *in vitro* and *in vivo* inhibition and interference with angiogenesis-mediated by *α*_v_*β*_3_ integrins^153^.

### 4.4 NPs and cytokine-mediated immunotherapy

Cytokines are proteins that function as communication mediators for the immune system, facilitating interactions through autocrine or paracrine secretion^154^. They play critical roles in anti-tumor strategies through their anti-proliferative, pro-apoptotic activities and promotion of cytotoxic effector cell recognition of tumor cells. Over the past few decades, numerous cytokines, including IL-2, IL-7, IL-12 CSF1R, and IL-6, have been effectively employed for cancer treatment^155^. However, the potential of cytokine-mediated immunotherapy has been restricted due to several limitations, such as inducing autoimmune responses, exhibiting high off-target phenomena, and prompting cytokine release syndrome (CRS) and vascular leakage syndrome due to their short half-lives, necessitating high dose administrations^156^. Due to their efficient targeting capabilities and dose control, NPs offer a promising avenue to circumvent these obstacles in cytokine application for tumor immunotherapy.

NP-mediated tumor immunotherapy can modulate the tumor's inflammatory response. For example, administering 20 mg/kg of titanium dioxide nanoparticles can stimulate M1 macrophages to release substantial amounts of pro-inflammatory cytokines and chemokines, whereas M2 macrophages produce large quantities of IL-10, thereby significantly inhibiting metastasis in hepatocellular and lung cancers^157^. NPs can serve as dedicated cytokine delivery platforms to boost tumor immune responses. One study encapsulated interleukin-12 into NPs and observed that compared to systemic NP delivery, IL-12 NP therapies led to reduced severe toxicity while retaining anti-tumor efficacy. This approach drove pro-inflammatory immune responses in immune cold tumors, resulting in a 30% complete survival rate in ovarian cancer. In addition, nanoparticles can modulate tumor immunotherapy by delivering investigational factor-related gene editing platforms^158^. For instance, LNPs modified with CD3 antibodies were loaded with plasmids of human interleukin-6 (IL-6) short hairpin RNA (shRNA) and CAR genes. These LNPs successfully transfected T cells post-intravenous injection, generating IL-6 knockdown CAR-T cells *in vivo*. These LNP-mediated CAR-T cells displayed anti-tumor effects comparable to conventional CAR-T cells and reduced IL-6 release and the incidence of cytokine release syndrome (CRS)^159^.

### 4.5 NPs-based cell therapies

**Figure 6:**
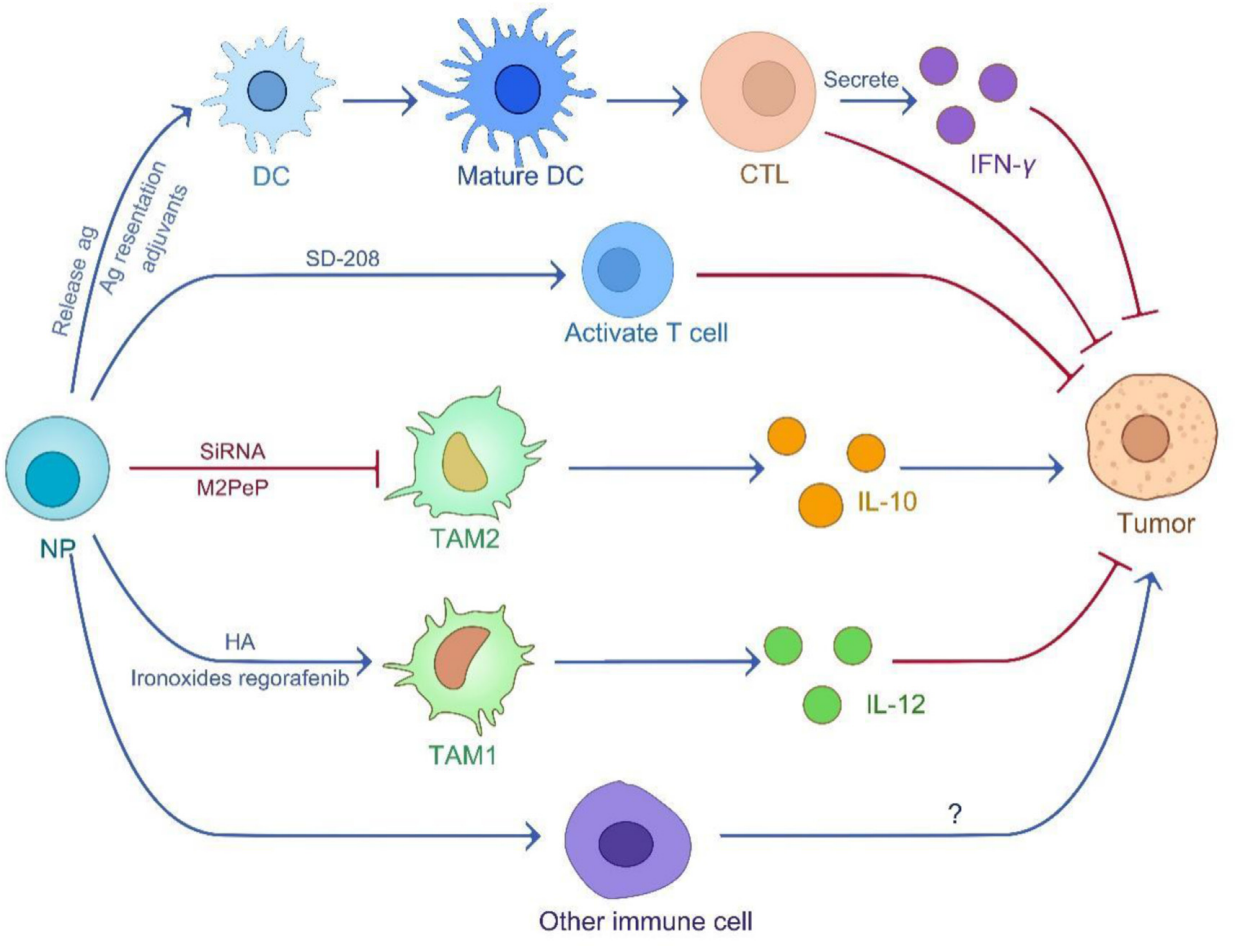
NPs combined with immune cell therapy to kill tumor cells strategy. NPs induce activation of dendritic cells through antigen presentation or release of antigen to inhibit tumor development. NPs can also carry siRNA, HA, and M2PeP to promote M1 polarization of tumor-associated macrophages or inhibit M2 polarization to reshape the tumor microenvironment and inhibit tumor growth. In addition, NPs loaded with SD-208 mediate tumor killing by directly activating T cells.

The innate immune cells primarily consist of natural killer (NK) cells, eosinophils, basophils, and phagocytes, including mast cells, neutrophils, monocytes, macrophages, and DCs that either directly kill tumor cells or trigger adaptive immune responses to restrict tumor progression^160^.The innate immune cells primarily consist of natural killer (NK) cells, eosinophils, basophils, and phagocytes, including mast cells, neutrophils, monocytes, macrophages, and DCs that either directly kill tumor cells or trigger adaptive immune responses to restrict tumor progression^161^. NPs can augment the body's immune response by activating tumor-killing immune cells and suppressing tumor-associated immune cells (Fig. 6).

#### 4.5.1 Targeted dendritic cells (DC)

Myeloid-derived DCs are crucial in antigen presentation and can be identified within blood plasma. Upon activation, these cells migrate to lymph nodes, forging the link between innate and adaptive immunity by interacting with T cells. The scarcity and impaired functionality of DCs in the TME pose significant challenges to the induction of adaptive immunity and the *in situ* initiation of T-cell responses. Therefore, augmenting the presence of DCs in the TME, their intra-tumor localization, and restoring their antigen-presenting capabilities are critical for effective tumor immunotherapy^162^^,^^163^.

Several studies have aimed to enhance tumor immunotherapy by targeting DCs and integrating specific ligands into NPs to bolster the activation and maturation of DCs. The research involved the creation of lipid-calcium-phosphate (LCP) NPs encapsulating the BRAF600E peptide along with the CpG oligodeoxynucleotide (ODN) adjuvant. The protective nature of the LCP NPs safeguarded the BRAF600E peptide and ODN molecules from enzymatic degradation by endogenous nucleases, enabled the targeting of DCs across physical barriers, and induced the maturation and activation of DCs. The results revealed significant alterations in the tumor tissue sections of mice treated with the BRAF peptide vaccine, including increased CD8 T cell counts and decreased Tregs and MDSCs compared to controls. Furthermore, 20% of mice treated with BRAF600E peptide NPs exhibited tumor-free survival, demonstrating the potential of NPs in enhancing antigen-specific T-cell responses through improved antigen targeting of DCs^164^. Several studies have aimed to enhance tumor immunotherapy by targeting DCs and integrating specific ligands into NPs to bolster the activation and maturation of DCs. The research involved the creation of lipid-calcium-phosphate (LCP) NPs encapsulating the BRAF600E peptide. An investigation by Liu et al.^165^ utilized magnetic Fe_3_O_4_@Ca/manganese oxide NPs loaded with OVA to efficiently deliver antigens to the cytoplasm of DCs, addressing the defects of poor *in vitro* activation, low antigen presentation level, reduced cell viability, and difficulty in targeting lymph nodes *in vivo* that are characteristic of conventional DC vaccines. In a magnetic field, the nanoparticles come into contact with cells. They released Mn^2+^, Ca^2+^, and antigens through hydrolysis in a mildly acidic environment, increasing IFN*β* and autophagy concentration. This process enabled the effective activation of DCs and enhanced antigen cross-presentation. It promoted the migration of activated DCs to lymph nodes, leading to a proliferation of T lymphocytes, prolonged memory T cell duration, and increased antibody levels. These changes underscore the potential of such strategies to boost the efficacy of tumor immunotherapy.

In addition to focusing on the activation function of NPs on DCs, NPs have been engineered as tracers to detect antigen targeting to DCs and migration. A study developed gold nanocages (AuNCs) loaded with the adjuvant monophospholipid A (MPLA), tyrosinase-related protein 2 (TRP2), and an antibody explicitly targeting the ligand CD11c, encapsulated within liposomes. This arrangement enhanced the biocompatibility and stability of the AuNCs while protecting the peptides from degradation and leakage. It was observed that almost all immature DCs of bone marrow origin (imBMDCs) displayed a distinct pattern of green fluorescence after incubation with the experimental group's Lipos-AuNC-MPLA-aCD11c-TRP2 for 1, 3, and 6 h, as opposed to the control group treated with Lipos-AuNC-MPLA-TRP2. The experimental group also revealed a significant accumulation of AuNCs in regional lymph nodes (RLNs) after subcutaneous injection, peaking at 12 h. These observations indicate the targeting ability and enhanced immune effect of the experimental group, reflected by the maturation of imBMDCs and the significantly reduced and smaller number of melanoma nodules in the lung metastasis model of melanoma B16–F10^166^.

#### 4.5.2 Targeting of tumor-associated macrophages

The TME comprises a diverse composition of cellular and molecular components, one common feature among solid tumors being the infiltration of bone marrow-derived mononuclear cells, predominantly monocytes, macrophages, and dendritic cells. Macrophages within the TME, commonly known as TAMs, exhibit dichotomous roles in cancer progression^167^. They can eliminate malignant cells, disrupt tumor vasculature, and invoke innate or adaptive lymphocyte-mediated antitumor responses. Conversely, they can endorse tumor growth and angiogenesis and suppress immune reactions through various mechanisms. This duality has made TAMs a focal point in current tumor immunotherapy research^168^. Strategies to target TAMs typically involve systemic anticancer drugs that can reverse the pro-tumor M2 TAM polarization and potentiate immunotherapeutic outcomes through macrophage depletion. It is widely accepted that within the TME, TAMs uptake NPs, providing a basis for nanoparticle-based immunotherapeutic strategies that involve coupling therapeutic agents to NPs for targeted TAM delivery^169^.

In a notable study, co-incubation of macrophages, iron oxide NPs, and cancer cells was conducted, which showed an upregulation of M1-associated markers (TNF*α* and CD86) in macrophages exposed to iron oxide NPs. Simultaneously, M2-associated markers (CD206 and IL10) were significantly downregulated. This was concurrent with an 11-fold surge in hydrogen peroxide production and a 16-fold increment in hydroxyl radical production relative to controls. Iron oxide NPs can suppress tumor growth by triggering a pro-inflammatory macrophage response^170^. Furthermore, chemotherapeutic agents like gemcitabine, 5-fluorouracil, and platinum drugs have been coupled to NPs to combat gastrointestinal tumors *via* the reversion of M2 TAM polarization^171^. Qian et al.^172^ engineered a novel dual-targeting nanoparticle (M2NP) specific for M2-like TAMs, loaded with scavenging receptor type B1 and M2-binding peptide (M2pep) to enhance TAM-targeted immunotherapy. When loaded with small interfering RNA (siRNA) targeting the anti-colony-stimulating factor-2 receptor on M1 NP, this nano complex exhibited higher TAM affinity than other macrophages and specifically blocked M2 survival signals. This study demonstrated that M2 NP treatment led to an elimination of 52% of M2 macrophages in treated mice, an 87% reduction in tumor volume, and increased survival, underscoring the therapeutic potential of such approaches cite a paper here.

#### 4.5.3 Other immune cells

NPs-based drug delivery platform facilitates a direct targeting approach in immune therapeutics, using chemokine gradients to actively recruit T cells to inflammation sites within tumors. Unlike conventional strategies of delivering immune drugs to tumor cells, this approach exploits lower immune stimulant concentrations to amplify the T cell-mediated antitumor^173^. For instance, a study demonstrated encapsulating SD-208 (a TGF*β* R1 inhibitor) in lactic-*co*-glycolic acid polymer NPs significantly improved survival in a colorectal cancer mouse model by rejuvenating T cell functionality. Despite limited reports employing such strategies in tumor immunotherapy, this may herald a new direction for future research in the field^174^.

Chimeric Antigen Receptor T-cell (CAR T-cell) therapy is an innovative and FDA-approved treatment for B-cell malignancies and multiple myeloma. The generation of these T cells primarily relies on viral vectors, but concerns about safety and high production costs limit the broad application of CAR T-cell therapy. In contrast, liposomes and polymeric NPs provide a promising alternative, offering efficient targeted gene transfection capabilities for T cells. This could address viral vector limitations and expand CAR T-cell therapy's applicability in future clinical settings^175^^,^^176^.

### 4.6 NP-mediated combination immunotherapies

Numerous contemporary studies suggest that combination therapies for tumors are superior to traditional monotherapies. Techniques such as PDT, PTT, and SDT are subjects of intense research for their potential in cancer treatment. Despite their potential, these therapies encounter several drawbacks, such as limited tumor-specific targeting, inadequate immune response for treating metastatic tumors, cytotoxicity of sensitizers, and collateral damage to healthy tissues. Incorporating NP-mediated immunotherapy can address these issues by conferring excellent targeting abilities to the sensitizers and enhancing overall tumor resistance *via* the amplification of immune responses. This section explores the advancements in NP-based engineering studies associated with amalgamating different therapeutic strategies with immunotherapy.

#### 4.6.1 Photodynamic therapy (PDT)

**Figure 7:**
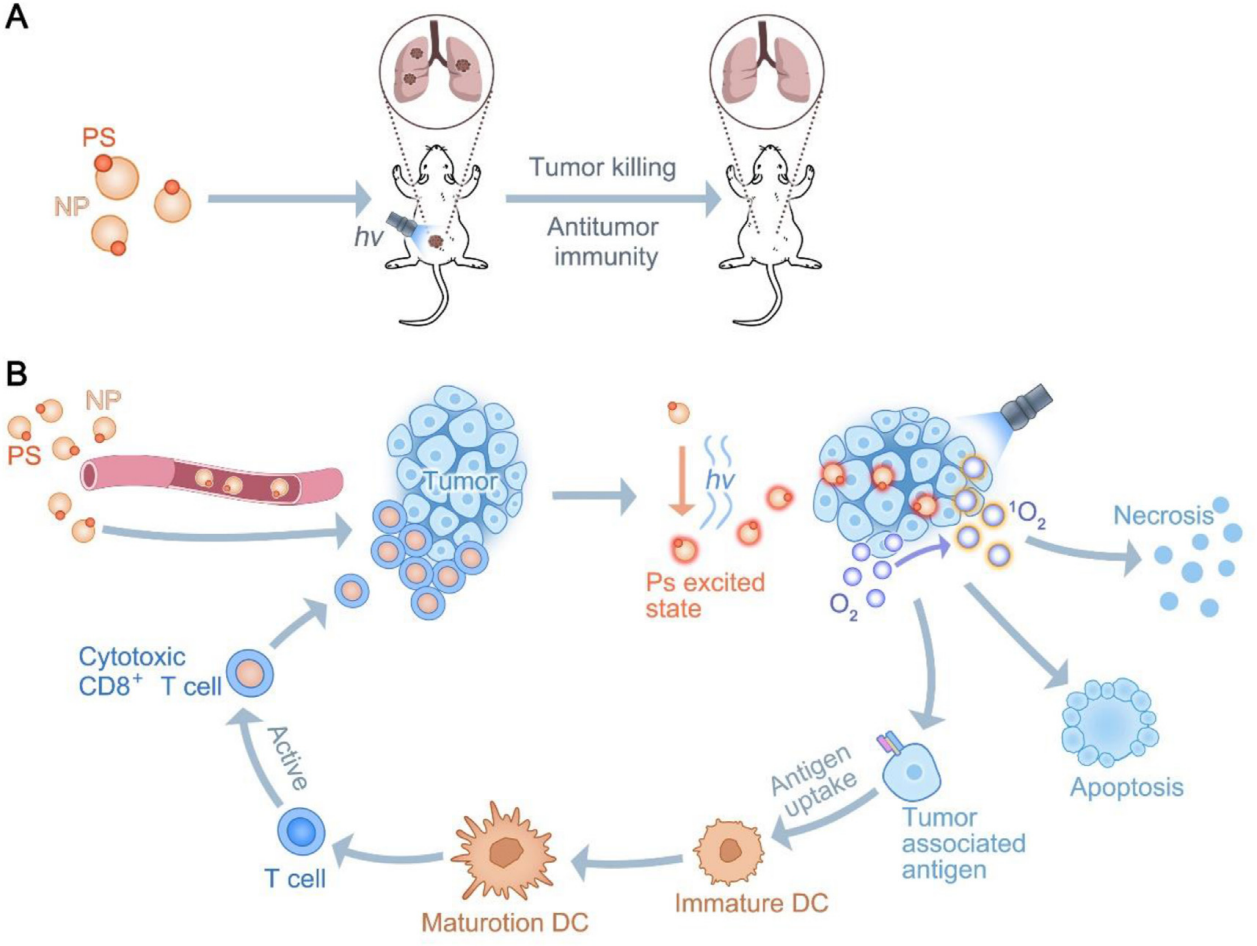
Mechanism of NPs-mediated PDT therapy. (A) Photodynamic therapy modality diagram. (B) Under light conditions, the photosensitiser combines with oxygen TME to produce ROS, thus producing a killing effect on tumor cells. Using apoptotic tumor cells as antigens, it activates the immune response of tumor cells, thus further enhancing the killing effect on tumor cells.

PDT is a non-invasive approach to induce cancer cell death *via* ROS generated by the bio–chemical interaction of photo-sensitizers (PSs) with oxygen TME under light exposure^177^. PDT combines direct necrosis and apoptosis for treating tumors, causing cellular cancer cell death and stunting tumor cell growth, along with the induction of extracellular heat shock protein 70 (HSP70) release from necrotic tumors to facilitate DCs activation and maturation^178^^,^^179^ (Fig. 7A). Although conventional PDT is associated with low toxicity, minimally invasive, and high response rates, the local stimulation it triggers is often inadequate to induce effective systemic immunity180. However, when combined with tumor immunotherapy, PDT can transcend this limitation. The low tumor-targeting capability of PSs and the collateral damage to healthy tissues have restricted the application of PDT for tumor immunotherapy. Various NPs have been developed to overcome these barriers to enhance the specific targeting of photosensitizers and protect hydrophobic photosensitizers from the aqueous environment^181^ (Fig. 7B). For example, a study developed a spherical nucleic acid (SNA) based on a PD-L1 aptamer, which consists of OXA encapsulated in a core of metal–organic framework nanoparticles and a dense shell of aptPD-L1 (denoted M@O-A), where M@O-A can specifically target and block tumor cells with high expression of PD-L1, and which, in the presence of light conditions, ROS and OXA, triggering ICD and augmenting immune therapies while attenuating immune-related adverse events (irAE)^181^.

#### 4.6.2 Photothermal therapy (PTT)

PTT, which capitalizes on the unique hypoxic and acidic TME that makes tumor cells more susceptible to heat than normal tissues, is emerging as a promising technique for tumor treatment^182^^,^^183^. Despite its potential, PTT has limitations, such as limited tumor penetration of near-infrared light, insufficient efficacy for distant metastatic and recurrent tumors, and a photo-bleaching effect that diminishes the thermal efficiency of small organic molecules^184^^,^^185^. Researchers have combined PTT with multiple immunotherapy tools, such as tumor vaccines, immune checkpoints, and immune adjuvants, with promising outcomes to overcome these drawbacks^186^. By capitalizing on the effects of enhanced permeability, permeability, and retention (EPR) and loading tumor-targeting ligands, NPs can enhance the efficacy of immune-boosted PTT^187^. For example, in one study, the photosensitizer NR840 and lactate oxidase (LOX) were assembled with targeted DSPE-PEG-cRGD. Under 808 nm laser irradiation, the thermal energy released by NR840 damaged tumor cells, promoted ICD, and recruited "immuno-thermal" tumors by secreting cytokines and subsequently, through a precise metabolic process, achieved photothermal ablation of 4T1 breast tumors and elicited a robust antitumor immune response. Accurate metabolic processes performed the photothermal ablation of 4T1 breast tumors, and a solid antitumor immune response was induced, which effectively prevented immune escape and eliminated spontaneous lung metastasis of breast cancer with the help of the excretion of immune-promoting factors and anti-programmed death ligand 1 (anti-PD-L1)^182^.

#### 4.6.3 Sonodynamic therapy (SDT) combined with immunotherapy

SDT leverages low-intensity ultrasound and a sono-sensitizer to generate cytotoxic ROS for cancer and antimicrobial therapy^188^. Given the reliable penetrating power of ultrasound, SDT is often employed for treating deep or hidden tumors^189^. When combined with immunotherapy, SDT can substantially enhance treatment efficacy by altering the immunosuppressive TME^190^. NPs-mediated tumor immunotherapy can amplify the synergistic effect of SDT and immunotherapy, leading to superior tumor treatment outcomes^191^. There is currently a consensus that the ideal treatment paradigm for cancer is not limited to the treatment of the primary tumor alone but should include the identification, inhibition, and removal of residual tumors at metastatic sites. Specifically, a study used chlorin e6 (Ce6) as an acoustic sensitizer to construct sPD-1 and Ce6 co-loaded NBs. sPD-1/Ce6, with the assistance of engineered nano-assisted sPD-1/Ce6, enables tumor cell-targeted delivery and localized release, which, on the one hand, induces cancer cell death under ultrasonication conditions by cavitation and acoustic wave effects, expanding the integrity and permeability of the vascular wall and the cellular membrane, in addition, driven by Ce6-induced SDT, sPD-1 increased the production of immune factors and the killing of NKs and CTLs, resulting in effective synergistic immunotherapy against HCCs^192^ (as shown in Table 2^180^^,^^181^^,^^185^^,^^189^^,^^191^^,^^192^).

**Table 2:** Mechanism of NPs combined with novel immunotherapy.

Type of NPs	Carry good	Disease treatment	NPs effect	Combination immunotherapy strategy	Ref.
Metal nanoparticles	Oxaliplatin (OXA) and aptPD-L1	Pancreatic	Enhanced tumor targeting	Nanoparticle PDT under light conditions down-regulates the TGF*β* signaling pathway, leading to reduced drug resistance, proliferation and migration of cancer cells. Meanwhile, pancreatic stellate cells (PSCs) were inactivated by PDT, hindering ECM secretion	180
Polymer NPs	NR840 and acid oxidase	4T1 breast cancer cells	Good biocompatibility and active tumor targeting ability	PLNR840 photothermal properties led to tumor cell damage and lactate depletion alleviated the abnormal metabolism of tumor cells, remodeling the immunosuppressive TME.	181
Au NPs	Immune adjuvant CpG oligodeoxynucleotide (ODN) and indoleamine 2,3-dioxygenase (IDO) inhibitor (NLG919)	Breast cancer	Tumor targeting, long drug cycle	CpG triggers tumor-specific immunity. Reversal of the immunosuppressive microenvironment mediated by photothermal conditioned IDO inhibitors.	185
Liposome	HMME and R837	4T1 breast cancer	Efficient intracellular uptake and low efflux	Acoustic sensitizers and imiquimod (R837) under ultrasound conditions trigger an immune response by promoting maturation of dendritic cells (DCs) and cytokine secretion to kill tumor cells	189
Metal nanoparticles	Ce6 and sPD-1	Hepatocellular carcinoma	Enhanced permeability and retention effects	Ultrasonication conditions treated with sPD-1 increased production of immune factors and killing of NKs and CTLs in response to Ce6-induced SDT drive	191
Mesoporous silica	Astragaloside III and Ce6	Colon cancer	Promote tumor drug penetration	As + Ce6@MSNs-PEG effectively activates NK cells and enhances the cytotoxicity of natural killer cells and CD8 T cells *in vivo* under light conditions	192

NPs, nanoparticles; PDT, photodynamic therapy; PD-1, programmed death 1; Ce6, chlorin e6; ECM, extracellular matrix; NK, natural killer cell; CTLs, cytotoxic T lymphocytes; TME, tumor micro-environment.

## 5 Rational selection of NPs as delivery vehicles for immunotherapy

Nanotechnology holds vast potential for enhancing drug delivery and treatment strategies, including tumor immunotherapy. Various attributes of nanomaterials, including their origin, dimensions, morphology, and surface charge characteristics, significantly influence their effectiveness in delivering immune-related drugs^193^.

Nanoparticles can be engineered into shapes, such as rods, spheres, cubes, and discs. The asymmetry of nanoparticle shapes can alter their tumbling motion within the bloodstream, thereby influencing tumor-targeting^194^. One study reported higher specific and lower nonspecific uptake of spheroidal nanoparticles versus rod-shaped vinyl nanoparticles in three breast cancer cell lines (BT-474, SK-BR-3, and MDA-MB-23)^195^. The size of the nanoparticle-based carrier system plays a crucial role in tumor immunotherapy. Particle size affects the clearance rate within the organism and influences hemodynamics. The movement of smaller molecules primarily occurs *via* free diffusion, whereas diffusion and convection processes regulate the transport of larger nanoparticles^196^. In a study, mice were injected with 10, 22, and 33 nm OVA-GNPs *via* the tail vein. The study revealed a size-dependent impact on DC function, with larger vector sizes improving OVA delivery to draining lymph nodes and enhancing CD10 T cell infiltration^197^.

Surface charge modification is another pivotal factor that can bolster nanoparticle-mediated tumor immunotherapy. Introducing charge can increase the number of therapeutic immune drug binding sites on the nanoparticle surface through electrostatic interactions. Linear polyethyleneimine, for example, is widely recognized as a cationic polymer capable of binding DNA and RNA and delivering them to the cytoplasm for therapeutic effect. However, cationic nanoparticles may be associated with acute systemic toxicity and nonspecific immunity, presenting a considerable barrier to their widespread utilization. Therefore, ongoing research is urgently needed to address toxicity and immunogenicity issues linked with cationic NPs^198^.

The material source of NPs also significantly impacts their therapeutic efficiency. For instance, membrane-encapsulated nanoparticles inherit properties from their parent cells due to their unique biofilm structure. As such, macrophage membrane-like nanoparticles exhibit superior immune evasion abilities compared to other nanoparticles. Tumor cell membrane-like nanoparticles demonstrate remarkable tumor-homing and targeting effects^199^. Metal-derived nanoparticles possess unique light scattering skills, making them ideal for tumor immunotherapy in combination with photothermal and photodynamic therapies. Gold nanoparticles have inherent immunogenic properties, enabling them to activate immune cells such as macrophages and dendritic cells. This leads to enhanced phagocytosis and increased secretion of immune-inflammatory cytokines^200^. For future nanoparticle designs intended for tumor immunotherapy, it is crucial to consider factors such as size, source material, charge profile, and shape. The key to successful tumor immunotherapy lies in developing and designing nanoplatforms that are biologically compatible and fit for practical research applications.

## 6 Prognostic analysis of diverse NPs combined with tumor immunotherapy

NPs have driven tumor immunotherapy due to their remarkable adaptability, excellent chemical versatility, superior biocompatibility, and sufficient drug-loading capacity. Here, we dissect the potential prognostic value of multiple NPs in combination with immunotherapy for tumor treatment.

Mesoporous silica: a study loaded purified protein derivatives of human tuberculin into mesoporous silica (PDD-MS) to activate T cells to kill tumor cells by increasing antigen uptake by DCs, and 80% of lung cancer mouse models were recurrence-free within 30 days after treatment^201^. Based on this study, the authors loaded calcium phosphate into PDD-MS to form the PPD-MS/CaP adjuvant delivery system. Post-treatment tumor recurrence risk challenge experiments found that after the second injection of Lewis lung cancer (LLC) cells, tumors developed after seven days in the PPD-MS and free PPD groups and after 11 days in the PPD-MS/CaP group^202^. A study by Wang et al.^203^ confirmed that MS itself triggers the TLR4/NF-*κ*B pathway in macrophages, driving the expression of T-cell recruitment chemokines, promoting tumor infiltration in CTL and causing immunogold tumor inflammation in mice treated with MS in a mouse model of hepatocellular carcinoma and colorectal cancer, with no risk of recurrence within ten days. The above data reveal that *via* MS itself, MS combined with immune drugs can improve the prognosis of tumor immunotherapy, and reasonable modifications of it can enhance this effect.

Liposomes: liposomal nanoparticles loaded with cyclic dinucleotides and nebulized (AeroNP-CDN) attenuated immunosuppression TME by reprogramming TAM from M2 to M1 phenotype and enhancing CD8 T-cell tumor infiltration. 40% of non-small cell lung cancer (LANSCLC) mouse models survived for more than 120 days after AeroNP-CDN treatment. The study by Matthias T stephan et al.^204^ clearly showed that liposome-encapsulated CAR-T therapy resulted in a doubling of breast cancer holo-survival and a 17-day improvement in survival compared to conventional cell-based therapies. In addition to directly mediating tumor immune killing, NPs can also modulate the level of memory cells in the body. In one study, synthetic-wrapped gemcitabine and R837 liposomes were applied to tumor treatment. The therapy achieved complete inhibition of spontaneous metastasis of tumor cells from the primary tumor to the lung and inhibited tumor growth for up to 20 days by locally producing memory T cells in a tumor recurrence challenge assay^205^.

Polymeric NPs: Zheng et al.^206^ developed a novel polypyrrole nanoparticle by coupling the near-infrared dye IRDye800CW with camptothecin (CPT) associated with hyaluronic acid (HA) shells (PPy@CPT-HA-IRDye800CW) to achieve PPT combined with immunotherapy applied to the treatment of breast cancer, which resulted in complete tumor eradication, no tumor recurrence within 24 days and a median survival time of more than 60 days in tumor-bearing mice. In contrast, a study by Zhang et al.^207^ used tumor cell lysates as a nano vaccine (TCLN) transplanted onto polydopamine nanoparticles to prepare an alginate hydrogel containing endostar applied to MC38 tumor-bearing mice, which killed tumor-cells-by-reducing-tumor-angiogenesis-expression-of-tumor-microenvironment-related cytokines (TMCs) and promoting the activity of CTLs. The survival study found that treatment with TCLN/EH resulted in complete tumor regression with a 62-day survival rate of 5.62% compared to a 0% survival rate in the EH group. An additional cRGD-modified cancer cell membrane (CM)-encapsulated calcium carbonate nanoparticles were designed to deliver interleukin-12 messenger RNA (IL-12 mRNA@cRGD-CM-CaCO_3_NPs), which was released from the nanoparticles in acidic tumor microcycles and translated into IL-12 in the cytoplasm, stimulating the proliferation and activation of CTLs and cytokine production to inhibit glioblast. Survival studies found that combined NPs of IL-12 mRNA@cRGD-CM-CaCO_3_ combined with ultrasound radiation treatment prolonged survival in mice and resulted in a 40% durable cure rate^208^. While Zhang et al.^209^ used ultrasound to encapsulate platelet membranes into nanoparticles encapsulated with the TLR agonist R848 polylactic acid (PLA), NPs-R848 increased tumor immune cell infiltration by promoting strong activation of APCs in draining lymph nodes (DLN) through the infiltration retention effect of NPs, and 28.6% survival in MC848 murine colon adenocarcinoma model after treatment with NPs-R848.

Other NPs: virus-like NPs, IPONs, Au-NPs, and other materials have been successfully developed for tumor immunotherapy, and these same NPs have a positive impact on the prognosis of tumors. For example, a study of Cd274 shRNA integration into the human tumor virus (HPV) L1 protein to generate a vaccine-based nanosystem enhanced the anticancer immune response by inhibiting tumor-specific PDL1 expression and preventing T-cell depletion. The nanosystem reduced tumor recurrence by 71% and extended progression-free survival by 67% in 4T1 breast cancer-bearing mice^210^(as shown in Table 3^200^^,^^202^^,^^205^, ^206^, ^207^, ^208^, ^209^, ^210^). Tian et al.^211^ designed the system of RNAi-M2pep-AuNPs to enhance tumor suppression in an *in situ* murine model of lung cancer by silencing VEGF mRNA in inflammatory tumor M2 macrophages and lung cancer cells; treatment with this system reduced the BALB/c tumor size of lung adenocarcinoma tumors by 95% and increased the survival rate of mice by 75%.

**Table 3:** NPs-tumor immunotherapy prognosis.

Nanoparticle type	Loaded cargo	Research subject	Mechanism of action	Prognosis	Ref.
Mesoporous silica	Human tuberculin purified protein derivatives	Lung cancer mice	Increased antigen uptake by DCs, activated T cells	Enhanced tumor recurrence risk challenge	200
Mesoporous silica	N/A	Mouse model of intestinal cancer	Drives expression of T cell recruitment chemokines and promotes tumor infiltration of CTL	No risk of recurrence in mice within 10 days	202
Polypyrrole nanoparticles	IRDye800CW, CPT and HA	Mouse model of breast cancer	Promotes tumor-specific CTL infiltration	Prolonged survival in hormonal mice	205
Polymeric NPs	TCLN	MC38 tumor-bearing mice tumor	Microenvironment-associated cytokines (TMCs) expression, elevated cytotoxic T lymphocytes activity	Prolonged survival of tumor-bearing mice	206
Polymeric NPs	Interleukin-12 mRNA	Glioblastoma	Proliferation and activation of cytotoxic T lymphocytes	Prolonged survival in tumor-bearing mice	207
PLA NPs	TLR agonist	MC848 murine colon adenocarcinoma model	Strong activation of APC in lymph nodes increases tumor immune cell infiltration	Improves survival in tumor-bearing mice	208
Virus-like NPs	Cd274 shRNA	Breast cancer tumor-bearing mice	Inhibits PDL1 expression and prevents T-cell failure	Reduces tumor recurrence	209
AuNPs	RNAi-M2pep	Lung adenocarcinoma tumor-bearing mice	Silencing VEGF mRNA increases	Survival in tumor-bearing mice	210

DCs, dendritic cell; CTL, cytotoxic T-lymphocyte; HA, hyaluronic acid; CPT, camptothecin; TCLN, tumor cell lysates nanovaccines; TLR, toll-like receptor; APC, antigen-presenting cell; PD-L1, programmed cell death 1 ligand 1; shRNA, short hairpin RNA; VEGF, vascular endothelial growth factor; N/A, not applicable.

Overall, NPs combined with immunotherapy strategies can break the bottleneck of conventional immunotherapy, enhance the effectiveness of tumor immunotherapy, and improve the prognosis of tumor immunotherapy (*e*.*g*., more prolonged survival, lower recurrence, lower risk of relapse challenge, etc.)

## 7 Conclusions and outlooks

The burgeoning field of nanomedicine over the past few decades holds substantial promise for advancing cancer immunotherapy. NPs offer significant advantages over traditional drug delivery platforms. They can be meticulously engineered to carry a wide array of therapeutic drugs, thereby enhancing the precision of immunotherapy, which low-targeting capabilities have traditionally hampered. Moreover, NPs can also address issues related to the limited immune response triggered by single-molecule drugs and the severe side effects associated with high-dose injections of immune drugs. The burgeoning field of nanomedicine over the past few decades holds substantial promise for advancing cancer immunotherapy. NPs offer significant advantages over traditional drug delivery platforms. They can be meticulously engineered to carry a wide array of therapeutic drugs, thereby enhancing the precision of immunotherapy, which low-targeting capabilities have traditionally hampered. Moreover, NPs can also address issues related to the limited immune response triggered by single-molecule drugs and the severe side effects associated with high-dose injections of immune drugs.

However, the application of NPs as immune drug carriers is not without its challenges:

1. While the high density of immune-related drug ligands confers superior tumor-targeting abilities, it also presents potential cytotoxicity risks to peripheral tissues. NPs represent a critical limitation that needs to be mitigated through effective strategies^212^.
2. Although the nanoscale dimensions of NPs provide excellent targeting capacity (*e*.*g*., crossing the BBB to target glioblastoma), they also lead to potential bioaccumulation in various organs like the lungs, liver, and kidneys through the digestive or respiratory tract. This inadvertent accumulation might result in unanticipated tissue damage^213^.
3. The biological clearance of NPs represents a dichotomy of challenges. On the one hand, rapid NP clearance by the body's mononuclear phagocytic system (MPS) and reticuloendothelial system (RES) drastically reduces drug concentration at target sites, thus diminishing the therapeutic effect. Several modifications, such as incorporating PEG, CD47, or cellular biofilm coatings, have been shown to mitigate this phagocytic clearance^214^. Conversely, NPs carrying therapeutic drugs can interact with various proteins or biological components, leading to systemic distribution and potential long-term retention of specific inert nanomaterials. Such bioaccumulation could obstruct tissue microcirculation, adversely affecting normal cells^215^.
4. Scalability and compliance with good manufacturing practice (GMP) regulations pose significant challenges for NP production. Large-scale production is crucial for transitioning nanomedicine to clinical application and commercialization^216^.
5. Despite extensive *in vitro* and animal studies, nanoparticle-mediated tumor immunotherapies have rarely transitioned to clinical tumor treatments, rendering NP-mediated clinical medicine relatively nascent. The journey towards clinical translation is fraught with difficulties, compounded by the fact that conventional subcutaneous tumor models, patient-derived xenograft (PDX) models, and genetically engineered mouse (GEM) models do not accurately mirror the natural progression of cancer in humans^217^.

Given the above limitations, to broaden the application of NPs in tumor immunotherapy as much as possible, we put forward the following suggestions:

1. Design: multidisciplinary intersection, design of relatively simplified NPs as much as possible without jeopardizing the therapeutic effect and by the safety principle.
2. Material selection: select NPs with high biocompatibility, low toxicity, and biodegradability as much as possible (exosomes may become a good choice).
3. Research subjects: choose animal models with predictive preclinical conditions for scientific research to better assess the clinical therapeutic effect and risk.
4. *In vivo* distribution issues: design suitable monoclonal antibodies, antibody fragments, peptides, growth factors, and other modifications of NPs to improve tumor cell targeting and reduce non-specific uptake. Combine with tissue and animal imaging technologies to accurately assess the distribution of NPs in tissue cells *in vivo.*
5. Deeply understand the physiological and pathological differences between individual patients and carefully assess the biological behavior of NPs in different individuals.

This review encompasses the myriad facets of NPs, focusing on their utilization in traditional immunotherapies and novel therapeutic strategies that combine NPs with sonodynamic therapy SDT, PTT, and PDT to enhance tumor immunotherapy. The potential of NPs-mediated tumor immunotherapy to augment cellular targeting and diminish drug off-target effects aligns well with the principles of precision medicine. However, extensive research is urgently needed to improve NP production, refine therapeutic strategies, and address safety concerns related to NP ingestion. Furthermore, elucidating strategies to enhance the efficacy of NPs while minimizing potential.

## Acknowledgments

This study was supported by grants from Karolinska Institute Network Medicine Global Alliance Collaborative Grant (C24401073, Sweden), China Postdoctoral Science Foundation (2021M703602), 10.13039/501100005047Natural Science Foundation of Liaoning Province (2022-BS-137, China).

## Author contributions

Xueqiang Peng: Funding acquisition, Writing – original draft. Jianjun Fang: Writing – original draft. Chuyuan Lou: Software. Liang Yang: Writing – original draft. Shaobo Shan: Formal analysis, Software. Zixian Wang: Conceptualization. Yutong Chen: Supervision. Hangyu Li: Conceptualization, Project administration, Supervision. Xuexin Li: Funding acquisition, Supervision.

## Conflicts of interest

The authors declare no conflicts of interest.
